# Malaria, malnutrition, and birthweight: A meta-analysis using individual participant data

**DOI:** 10.1371/journal.pmed.1002373

**Published:** 2017-08-08

**Authors:** Jordan E. Cates, Holger W. Unger, Valerie Briand, Nadine Fievet, Innocent Valea, Halidou Tinto, Umberto D’Alessandro, Sarah H. Landis, Seth Adu-Afarwuah, Kathryn G. Dewey, Feiko O. ter Kuile, Meghna Desai, Stephanie Dellicour, Peter Ouma, Julie Gutman, Martina Oneko, Laurence Slutsker, Dianne J. Terlouw, Simon Kariuki, John Ayisi, Mwayiwawo Madanitsa, Victor Mwapasa, Per Ashorn, Kenneth Maleta, Ivo Mueller, Danielle Stanisic, Christentze Schmiegelow, John P. A. Lusingu, Anna Maria van Eijk, Melissa Bauserman, Linda Adair, Stephen R. Cole, Daniel Westreich, Steven Meshnick, Stephen Rogerson

**Affiliations:** 1 Department of Epidemiology, UNC-Chapel Hill, Chapel Hill, North Carolina, United States of America; 2 Department of Obstetrics and Gynaecology, Edinburgh Royal Infirmary, Edinburgh, United Kingdom; 3 Department of Medicine at the Doherty Institute, The University of Melbourne, Parkville, Victoria, Australia; 4 UMR216-MERIT, French National Research Institute for Sustainable Development (IRD), Paris Descartes University, Paris, France; 5 Unite de Recherche Clinique de Nanoro, Institut de Recherche en Sciences de la Santé-DRO, Bobo-Dioulasso, Burkina Faso; 6 Departement de Recherche Clinique, Centre Muraz, Bobo-Dioulasso, Burkina Faso; 7 Medical Research Council Unit, The Gambia; London School of Hygiene and Tropical Medicine, London, United Kingdom; 8 Worldwide Epidemiology, GlaxoSmithKline, Uxbridge, United Kingdom; 9 Department of Nutrition and Food Science, University of Ghana, Legon, Accra, Ghana; 10 Department of Nutrition, University of California, Davis, California, United States of America; 11 Department of Clinical Sciences, Liverpool School of Tropical Medicine, Liverpool, United Kingdom; 12 Malaria Branch, Division of Parasitic Diseases and Malaria, Center for Global Health, Centers for Disease Control and Prevention, Atlanta, Georgia, United States of America; 13 Kenya Medical Research Institute (KEMRI)/ Centre for Global Health Research, Kisumu, Kenya; 14 Malaria and Neglected Tropical Diseases, Center for Malaria Control and Elimination, PATH, Seattle, Washington, United States of America; 15 Malawi-Liverpool-Wellcome Trust Clinical Research Programme, Blantyre, Malawi; 16 School of Public Health and Family Medicine, College of Medicine, University of Malawi, Blantyre, Malawi; 17 Center for Child Health Research University of Tampere School of Medicine and Tampere University Hospital, Tampere, Finland; 18 Walter and Eliza Hall Institute, Parkville, Victoria, Australia; 19 Institute for Glycomics, Griffith University, Gold Coast, Queensland, Australia; 20 Centre for Medical Parasitology, Depart. Of Immunology and Microbiology, Faculty of Health Science, University of Copenhagen, Copenhagen, Denmark; 21 National Institute for Medical Research, Tanga Centre, Tanga, Tanzania; 22 Department of Pediatrics, Division of Neonatal-Perinatal Medicine, School of Medicine, UNC-Chapel Hill, Chapel Hill, North Carolina, United States of America; 23 Department of Nutrition, UNC-Chapel Hill, Chapel Hill, North Carolina, United States of America; Mahidol-Oxford Tropical Medicine Research Unit, THAILAND

## Abstract

**Background:**

Four studies previously indicated that the effect of malaria infection during pregnancy on the risk of low birthweight (LBW; <2,500 g) may depend upon maternal nutritional status. We investigated this dependence further using a large, diverse study population.

**Methods and findings:**

We evaluated the interaction between maternal malaria infection and maternal anthropometric status on the risk of LBW using pooled data from 14,633 pregnancies from 13 studies (6 cohort studies and 7 randomized controlled trials) conducted in Africa and the Western Pacific from 1996–2015. Studies were identified by the Maternal Malaria and Malnutrition (M3) initiative using a convenience sampling approach and were eligible for pooling given adequate ethical approval and availability of essential variables. Study-specific adjusted effect estimates were calculated using inverse probability of treatment-weighted linear and log-binomial regression models and pooled using a random-effects model. The adjusted risk of delivering a baby with LBW was 8.8% among women with malaria infection at antenatal enrollment compared to 7.7% among uninfected women (adjusted risk ratio [aRR] 1.14 [95% confidence interval (CI): 0.91, 1.42]; *N* = 13,613), 10.5% among women with malaria infection at delivery compared to 7.9% among uninfected women (aRR 1.32 [95% CI: 1.08, 1.62]; *N* = 11,826), and 15.3% among women with low mid-upper arm circumference (MUAC <23 cm) at enrollment compared to 9.5% among women with MUAC ≥ 23 cm (aRR 1.60 [95% CI: 1.36, 1.87]; *N* = 9,008). The risk of delivering a baby with LBW was 17.8% among women with both malaria infection and low MUAC at enrollment compared to 8.4% among uninfected women with MUAC ≥ 23 cm (joint aRR 2.13 [95% CI: 1.21, 3.73]; *N* = 8,152). There was no evidence of synergism (i.e., excess risk due to interaction) between malaria infection and MUAC on the multiplicative (*p* = 0.5) or additive scale (*p* = 0.9). Results were similar using body mass index (BMI) as an anthropometric indicator of nutritional status. Meta-regression results indicated that there may be multiplicative interaction between malaria infection at enrollment and low MUAC within studies conducted in Africa; however, this finding was not consistent on the additive scale, when accounting for multiple comparisons, or when using other definitions of malaria and malnutrition. The major limitations of the study included availability of only 2 cross-sectional measurements of malaria and the limited availability of ultrasound-based pregnancy dating to assess impacts on preterm birth and fetal growth in all studies.

**Conclusions:**

Pregnant women with malnutrition and malaria infection are at increased risk of LBW compared to women with only 1 risk factor or none, but malaria and malnutrition do not act synergistically.

## Abstract 2

In a meta-analysis using individual participant data, Jordan Cates and colleagues explore potential interactions between malaria and nutrition during pregnancy and their associations with low birthweight.

## Author summary

**Why was this study done?:**

- More than 125 million pregnant women are at risk of malaria in pregnancy annually, producing detrimental effects on maternal, newborn, and infant health.
- Maternal undernutrition is estimated to be responsible for 800,000 newborn deaths annually.
- Prior evidence from 4 small studies indicated that the harmful impact of malaria on fetal growth and birthweight (BW) may depend upon the macronutrient nutritional status of the mother.
- If malaria and maternal undernutrition have synergistic negative impacts on pregnancy outcomes, interventions targeted to high-risk women might provide substantial public benefit.
- The present study provides a robust assessment of potential malaria–nutrition interactions in pregnancy and overcomes size and methodological limitations of earlier exploratory studies.

**What did the researchers do and find?:**

- We present a large, pooled analysis of individual participant data from 13 studies conducted in sub-Saharan Africa and the Western Pacific investigating the interaction between maternal malaria infection and malnutrition on the risk of low birthweight (LBW) and reduced mean BW.
- The findings suggest that women who are both infected with malaria and malnourished are at greater risk of LBW than their uninfected, well-nourished counterparts.
- However, the study found no conclusive evidence of interaction between the 2, i.e., the impact of malaria on BW was independent of the macronutrient nutritional status of the mother.
- Subgroup analyses did find that studies conducted just in Africa had slight evidence of interaction, but this was not consistent throughout all analyses.

**What do these findings mean?:**

- Although there was no overall evidence of malaria–nutrition interactions, more than 1 in 3 pregnant women suffered from malaria and/or undernutrition, emphasizing the importance of joint approaches to decrease maternal malaria and improve nutrition to minimize adverse pregnancy outcomes.

## Introduction

Annually, over 20 million infants are born low birthweight (LBW; <2,500 g), predominantly in low- and middle-income countries (LMICs) [1]. LBW can have negative impacts on neonatal mortality and childhood neurological, metabolic, and physical development [2]. The World Health Organization (WHO) has set a Global Nutrition Target of 30% reduction in LBW by 2025 [1].

One preventable cause of LBW in LMICs is maternal malaria infection [2,3]. Its prevalence remains high, despite targeted malaria prevention programs [2]. Annually, 125 million pregnant women are at risk for malaria [4]. The predominant species, *Plasmodium falciparum*, sequesters in the placenta, causing LBW through fetal growth restriction (FGR) and preterm delivery [2]. Prior estimates from Africa suggest that malaria infection doubles the risk of LBW [2,4]. The prevention of malaria infection during pregnancy remains a public health priority.

Another modifiable risk factor for impaired fetal growth is maternal malnutrition, specifically undernutrition [5]. Up to 20% of African women of reproductive age are undernourished [5–7]. Maternal protein-energy-fat (macronutrient) and micronutrient reserves and dietary consumption influence fetal growth. Micronutrient deficiencies are difficult and costly to assess; therefore, anthropometrics are commonly used as sensitive but nonspecific indicators of protein reserves, fat stores, and malnutrition more broadly [7].

Recent evidence indicates that the relationship between malaria infection and LBW may depend upon the mother’s nutritional status [8]. Studies in Papua New Guinea (PNG) and Benin found inconsistent evidence of modification of the malaria infection–LBW relationship by maternal anthropometric status, but studies from Kenya and the Democratic Republic of the Congo (DRC) reported significant modification [9–12]. Notably, in the DRC, the risk of FGR associated with malaria infection was 2 to 8 times higher among malnourished women [11]. Malaria infection and malnutrition may act along similar physiological pathways by affecting placental development and nutrient transfer [2,4,5].

To date, work on this potential interaction has been limited to 4 studies, with only 1,318 pregnant women from Africa and 1,369 pregnant women from PNG. Not only were these studies somewhat inconsistent in their findings, but their interpretation is hindered by relatively small sample sizes, and their findings may not be generalizable to other malaria-endemic countries. The objective of this study was to investigate the putative interaction between maternal malaria infection and malnutrition in relation to birthweight (BW) using a large, pooled dataset of 14,633 live birth pregnancies from women participating in 13 studies conducted in multiple LMICs. We hypothesized that there would be a synergistic interaction, such that the observed joint effect of being both infected with malaria and malnourished would be greater than expected if considering each exposure independently.

## Methods

### Study population

We used data from 14,633 singleton live birth pregnancies from women participating in 13 studies conducted from 1996 to 2015 in 8 African countries and the Western Pacific (PNG) as part of the Maternal Malaria and Malnutrition (M3) initiative [9,11,13–24]. The M3 initiative has been described in detail previously [25]. Briefly, the M3 initiative is a collaboration with the Malaria in Pregnancy Consortium (MiPc) and affiliated malaria and nutrition researchers who agreed to pool resources to improve the understanding of malaria–nutrition interactions. A convenience sampling approach was taken to obtain eligible studies identified by researchers within the MiPc, and inclusion of studies for the individual participant data meta-analysis stopped 1 January 2016. Studies were eligible if they were an observational study or randomized controlled trial conducted between 1996 and 2015 enrolling pregnant women during pregnancy with follow-up through delivery and they met the following criteria: ethical approval allowed for secondary analyses and data sharing, malaria was endemic in the area with medium to high transmission, assessment of malariometric indices (light microscopy [LM] and/or rapid diagnostic tests [RDT]) at enrollment/first antenatal care visit (ANC), assessment of anthropometric indicators at enrollment (mid-upper arm circumference [MUAC] and/or body mass index [BMI]), and assessment of infant weight within 24 hours postpartum or within 7 days of birth if timing of weight measurement data was available. Data was shared by each individual study using a standardized data transfer file. Participating studies had been undertaken for a range of objectives, including investigation of the mechanisms leading to LBW as a result of malaria, evaluation of antimalarial interventions during pregnancy such as intermittent preventive therapy during pregnancy (IPTp) or insecticide-treated bed nets (ITN), or the assessment of the potential of nutritional supplementation during pregnancy to improve birth outcomes (S1 Table). All studies received approval by their local ethics board and obtained informed consent from all participants. The prospective protocol for the IPD analysis is included in the supplemental text (S2 Text).

### Outcomes and exposures

The main outcome measure was BW, analyzed both continuously and dichotomized at 2,500 grams (LBW) [1]. Ten studies used digital scales to weigh newborns, 2 studies used spring or digital scales, and 1 study used a hanging weighing scale (S2 Table). Weights measured after 24 hours (13% of weights) were adjusted using a cubic regression model to account for weight changes in the first week of life [26]. Among 9 studies with ultrasound-dated gestational age, we considered 2 secondary outcomes: small for gestational age (SGA; a BW less than the 10^th^ percentile of the INTERGROWTH-21^st^ reference) and preterm birth (PTB; gestational age less than 37 weeks) [27].

Diagnostics for malaria were collected at study enrollment and at delivery. For the interaction analyses, we chose to focus on malaria infection at enrollment instead of at delivery for 2 reasons. First, from a public health perspective, if there was interaction at the time of study enrollment, this might help inform future interventions that could be implemented during antenatal care. Second, it has been hypothesized that malaria infection and malnutrition may act along similar physiological pathways to alter fetal growth by decreasing maternal–fetal oxygen transfer and reducing uteroplacental blood flow; 2 mechanisms that would be altered earlier in pregnancy versus at delivery. At study enrollment, we defined malaria based on LM examination of a Giemsa-stained peripheral blood smear or a RDT for malaria antigen [28]. At delivery, we defined malaria based on peripheral or placental LM or placental histology (active or past infection). Given the uncertain impact of submicroscopic infections on LBW and the variation in the availability of polymerase chain reaction (PCR) diagnostics across studies, we excluded PCR results [29]. In sensitivity analyses, we explored alternative definitions of malaria, including any PCR results and “any malaria,” defined as a positive LM, RDT, or PCR at enrollment, delivery, or during pregnancy (in 5 studies with repeat malaria diagnostics throughout pregnancy).

The primary measure of maternal malnutrition was low MUAC at enrollment, dichotomized at 23 cm [7]. MUAC changes little over pregnancy, making it a useful measure of malnutrition [7]. Since some studies did not measure MUAC, we used BMI as a secondary measure of malnutrition. According to WHO, a prepregnancy BMI <18.5 kg/m^2^ is predictive of adverse birth outcomes [30]. BMI at enrollment was used to estimate prepregnancy BMI by adjusting maternal weight measured in the second/third trimesters using a cubic regression model to account for gestational weight gain [30]. Low adjusted-BMI was defined as values under 18.5 kg/m^2^. As the correlation between BMI and MUAC is not perfect, indicators were analyzed separately [7]. The reason for dichotomizing MUAC and BMI was 2-fold. First, cutoffs are endorsed by WHO, are clinically easier to use, and are commonly used in the current literature to define undernutrition [7]. Second, while continuous exposures can be assessed in interaction models, interpretation is difficult, as the interaction estimates vary according to the levels of the exposures being compared and can vary in directionality as well [31].

### Risk of bias assessment

We developed a checklist of study characteristics for each of the included individual studies to assess the risk of bias for the main evaluation of the interaction between malaria infection and maternal malnutrition on BW. Criteria were specific to the research question and were informed by the Newcastle-Ottawa Scale, Downs and Black instrument, and the Meta-Analysis of Observational Studies in Epidemiology checklist [32–34]. For each included study, we evaluated the individual study publications or contacted individual study collaborators to identify the following items to categorize studies as being either at lower or higher risk of bias: participant retention rate (<75% versus ≥75%), measurement of important confounders (maternal age, gravidity, rural versus urban residence, HIV infection, and anemia at enrollment), clearly described measurement of malaria parasitemia, measurement of MUAC and/or BMI, >80% of BWs measured using electronic scale with known precision ≤20 g, and >80% BWs measured within 24 hours. Studies were defined as at lower risk of bias if every item was determined to be at a lower risk of bias.

### Statistical analysis

We analyzed maternal malaria infection and malnutrition as coprimary exposures and assessed malnutrition as a modifier of the malaria–LBW relationship. While effect measure modification (EMM) assesses how the effect of 1 exposure varies across strata of another variable, interaction analyses assess the joint effects of 2 exposures [35]. We performed both interaction and EMM analyses; however, in the context of this work, interaction is preferable to EMM because interventions for both malaria infection and malnutrition might prevent LBW.

There are 2 commonly employed approaches for handling individual pooled data, a 1-stage and a 2-stage approach, although there is no consensus as to which approach is preferable [36–38]. We employed a 2-stage approach, as it is generally considered more easily interpretable and allows the investigator to visually present forest plots and quantify statistical heterogeneity [36]. We examined the consistency of results with a 1-stage approach, fitting a generalized mixed model with random intercepts and slopes. Study-specific risk ratios (RRs) and mean BW differences were calculated using linear and log-binomial regression models controlling for confounding using inverse probability of treatment weights (IPTW) truncated at the 1st and 99th percentiles. A minimally sufficient set of confounders was identified using a directed acyclic graph based upon background knowledge of covariate relationships [39]. We identified confounders for both malaria infection and malnutrition relative to LBW since we were analyzing them as coprimary exposures. Confounders for the relationship between malaria infection at enrollment and LBW included maternal age, gravidity, rural versus urban residence, malnutrition (MUAC when available, otherwise BMI), and HIV infection. Because malaria infection is a cause of anemia, the latter was considered a mediator and not a confounder. We explored modification of the effect of malaria infection at enrollment on LBW by maternal gravidity and doses of intermittent preventive therapy (IPTp) received. When assessing malaria infection at delivery, anemia at enrollment and the number of IPTp doses were considered additional confounders. Confounders for the malnutrition–LBW relationship included maternal age, gravidity, rural versus urban residence, anemia at enrollment, and HIV infection. Partially missing data were imputed using multivariate normal multiple imputation (S1 Text) [40]. We calculated interaction estimates using a product term in the multiplicative and additive model for LBW and the additive model for mean BW [35]. These estimates reflect whether the effect of exposure to both malaria infection and malnutrition exceeds the product (or sum) of the effects of each exposure considered separately, defined as synergy. A product term greater than 1 on the multiplicative scale or greater than 0 on the additive scale is indicative of synergistic interaction between malaria infection and malnutrition.

Study-specific estimates were pooled using DerSimonian and Laird restricted maximum likelihood method random-effects models [41]. When *τ*^2^, the estimated variance of the random-effects distribution, was greater than 0, we calculated 95% population effects intervals (PEI), which incorporate the estimated variance between studies [41]. If *τ*^2^ equaled 0, the random-effects model was interpreted as a fixed-effects model. We decided a priori to evaluate the modification of the results by time period (before versus after 2008) due to changes in antimalarial recommendations, study type (trial/cohort), location (Africa/Western Pacific), and the study-level prevalence of malaria infection at study enrollment and delivery based on the individual study data, using meta-regression. We further decided post hoc to conduct a sensitivity analysis for the interaction analyses restricted to adolescent women.

## Results

**Fig 1:**
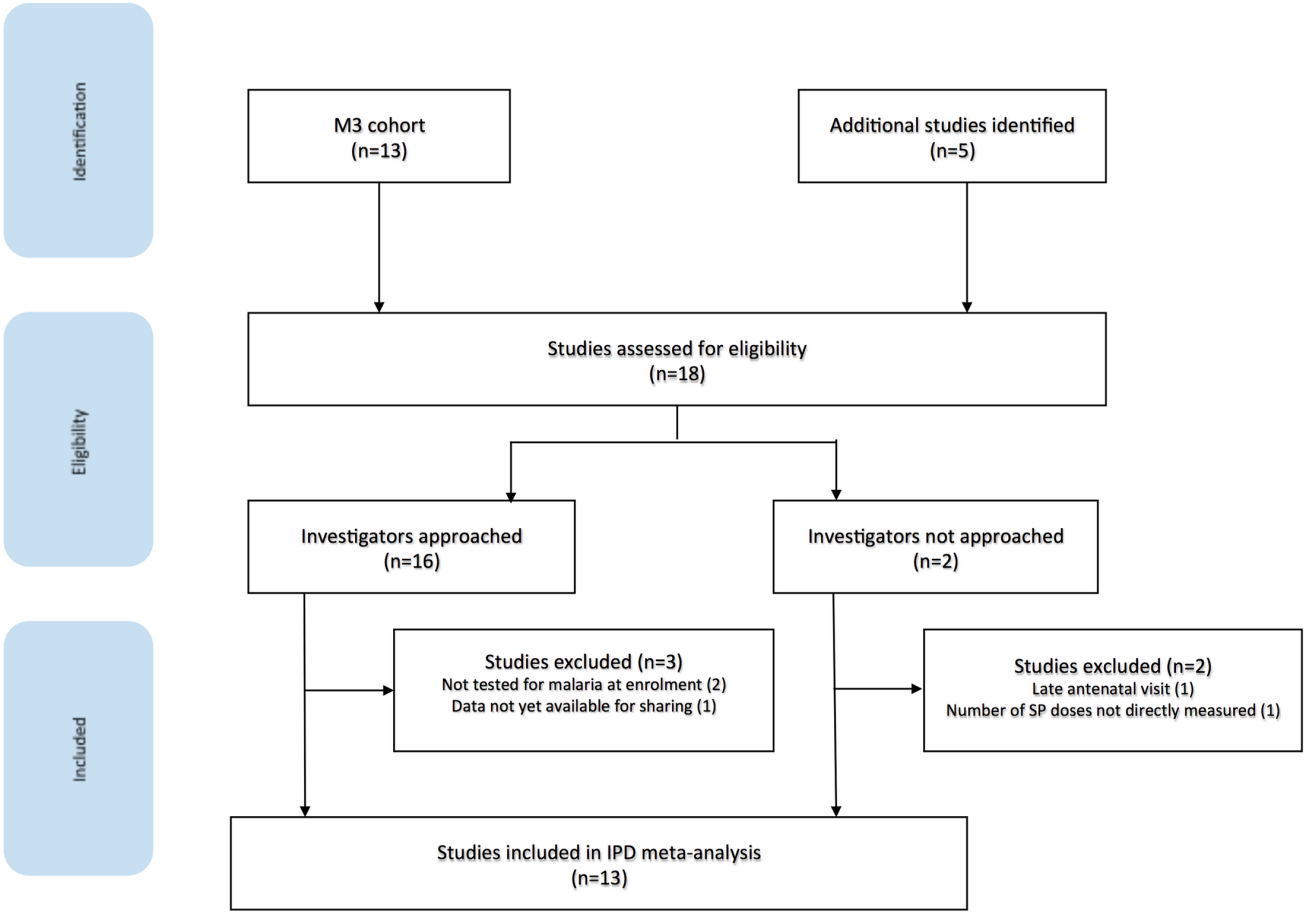
Flow diagram of studies included in the individual participant meta-analysis of the interaction between malaria infection and maternal malnutrition on birthweight. M3, Maternal Malaria and Malnutrition Initiative.

Using a convenience sample approach, a total of 18 studies were considered for inclusion by the time of our inclusion cutoff date (1 January 2016), of which 13 were included in the pooled analysis (Fig 1). We excluded 5 studies: 2 studies did not assess malaria at antenatal enrollment [42,43], 1 study had data that were not yet available for inclusion [44], 1 recruited women comparatively late in pregnancy [10], and 1 had not directly measured the number of sulfadoxine-pyrimethamine (SP) doses given for IPTp [45]. Following the cutoff date, 5 further studies were identified, of which 4 could be eligible with a collective sample size of 3,528 pregnant women (S3 Table) [46–50].

### Study population characteristics

**Fig 2:**
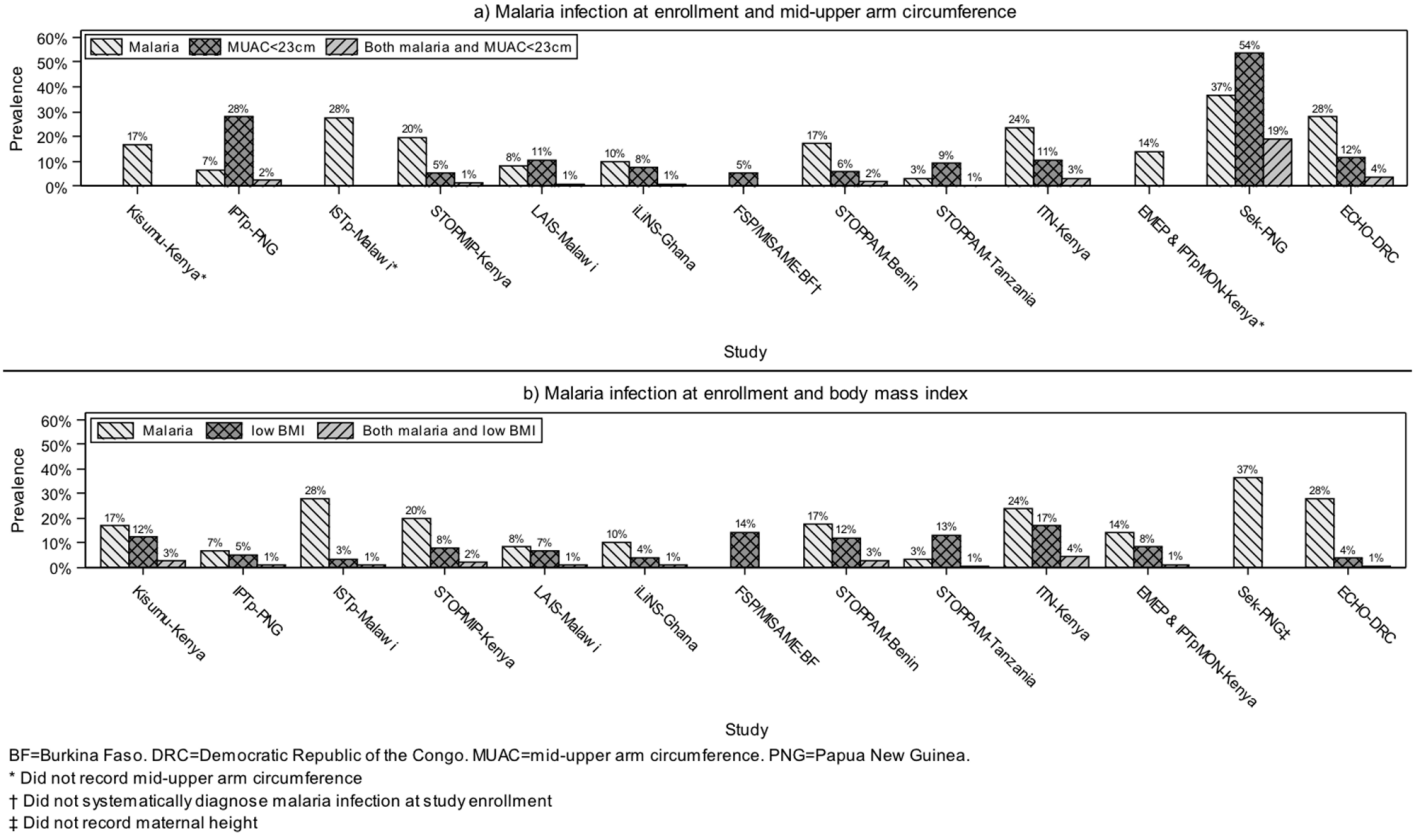
Prevalence of malaria infection at enrollment, malnutrition (mid-upper arm circumference [MUAC] < 23 cm or body mass index [BMI] < 18.5 kg/m^2^), and joint malaria infection and malnutrition among 14,633 live birth pregnancies from women participating in studies (years 1996–2015) included in the Maternal Malaria and Malnutrition (M3) initiative.

Twenty-five percent of the pooled dataset comprised adolescent women aged 19 or younger. The trimester at enrollment, anemia prevalence, gravidity distribution, area of residence, and HIV prevalence varied across studies (Tables 1 and 2). The prevalence of malaria infection at enrollment, malaria infection at delivery, low MUAC, and joint malaria infection at enrollment and low MUAC also varied by study (Fig 2 and S5 Fig). Among 8,152 women with both measurements, only 2% had both low MUAC and malaria infection at enrollment. The prevalence of malaria infection among women with low MUAC was 16%, compared to 12% among well-nourished women (*p* = 0.0005). The prevalence of low BMI varied across studies and was different from, although correlated with, the prevalence of low MUAC (*χ*^2^ *p* < 0.0001; S1 Fig). The joint prevalence of malaria infection at enrollment and low BMI was also 2%. Of all 14,633 women, 35% were infected with malaria at either enrollment or delivery or had low MUAC or BMI. The prevalence of LBW was 9% (range 5% to 15% among studies). Among 9 studies with ultrasound-dated gestational age, the prevalence of SGA was 19% (range 13% to 25%), and the prevalence of PTB was 11% (range 3% to 20%).

**Table 1:** The characteristics of women included in the Maternal Malaria and Malnutrition (M3) initiative from the following 6 out of 13 M3 studies: Kisumu-Kenya, IPTp-PNG, ISTp-Malawi, STOPMIP-Kenya, LAIS-Malawi, and iLiNS-Ghana.

	Kisumu-Kenya (*N* = 3,388)	IPTp-PNG (*N* = 1,943)	ISTp-Malawi (*N* = 1,602)	STOPMIP-Kenya (*N* = 1,203)	LAIS-Malawi (*N* = 1,190)	iLiNS-Ghana (*N* = 1,068)
Study enrollment (years)	1996–2001	2009–2013	2011–2013	2012–2015	2003–2006	2009–2012
Maternal age	20 (18–24)	24 (20–28)	21 (18–26)	22 (19–27)	24 (20–29)	26 (22–30)
Gravidity						
1 (Primi-)	1,656 (49)	966 (50)	542 (34)	403 (34)	267 (22)	349 (33)
2 (Secundi-)	748 (22)	494 (21)	448 (28)	237 (20)	213 (18)	351 (33)
3+ (Multi-)	984 (29)	573 (29)	612 (38)	563 (47)	710 (60)	368 (34)
Trimester*						
1	0 (0)	72 (4)	0 (0)	21 (2)	0 (0)	103 (10)
2	0 (0)	1,780 (92)	1,585 (99)	991 (82)	1,190 (100)	881 (82)
3	3,388 (100)	91 (5)	17 (1)	191 (16)	0 (0)	81 (8)
Missing GA	0 (0)	0 (0)	0 (0)	0 (0)	0 (0)	3 (0)
Anemic^†^						
Yes	2,548 (75)	1,348 (69)	533 (33)	591 (49)	459 (39)	305 (29)
No	808 (24)	512 (26)	1,069 (67)	612 (51)	731 (61)	763 (71)
Missing	32 (1)	83 (4)	0 (0)	0 (0)	0 (0)	0 (0)
HIV						
Yes	810 (24)	–	0 (0)	0 (0)	144 (12)	0 (0)
No	2,560 (76)	–	1,602 (100)	1,203 (100)	931 (78)	1,059 (99)
Missing	18 (1)	1,943 (100)	0 (0)	0 (0)	115 (10)	9 (1)
Area of Residence						
Rural	722 (21)	1,185 (61)	1,590 (99)	1027 (85)	1,190 (100)	0 (0)
Urban	2,666 (77)	758 (39)	10 (1)	169 (14)	0 (0)	1,068 (100)
Missing	0 (0)	0 (0)	2 (0)	7 (1)	0 (0)	0 (0)
IPTp doses	0 (0–0)	1 (1–3)	4 (3–4)^‡^	2 (1–3)^‡^	4 (2–4)	–
Bed net ownership						
Yes	–	1,798 (93)	327 (20)	681 (57)	877 (74)	–
No	–	145 (7)	1,275 (80)	522 (43)	313 (26)	–
Missing	3,388 (100)	0 (0)	0 (0)	0 (0)	0 (0)	1,068 (100)

Categorical variables are expressed as N (%) and continuous variables are expressed as median (IQR). A dash indicates information on particular factor was not assessed in parent study.

* Based on ultrasound if measured, otherwise based on Ballard’s score or symphysis-pubis fundal height (SFH). When using SFH, to adjust for misclassification in the first trimester, a fundal height <7 cm was defined as first trimester, while SFH < 28 cm was defined as second trimester, and SFH ≥ 28 cm was defined as third trimester.

^†^ Anemic = hemoglobin <11 g/dL of venous blood, if available, or hematocrit <33% in the first and third trimesters and less than 10.5 g/dL and 32%, respectively, for the second trimester.

^‡^ Excluding women randomized to the intermittent screening and treatment group.

GA, gestational age; iLiNS, International Lipid-Based Nutrient Supplements; IPTp, intermittent preventive treatment in pregnancy; ISTp, intermittent screening for malaria infection during pregnancy; LAIS, Lungwena Antenatal Intervention Study; M3, Maternal Malaria and Malnutrition; PNG, Papua New Guinea; STOPMIP, strategies to prevent malaria infection during pregnancy.

**Table 2:** The characteristics of women included in the Maternal Malaria and Malnutrition (M3) initiative from the following 7 out of 13 M3 studies: FSP/MISAME-BF, STOPPAM-Benin, STOPPAM-Tanzania, ITN-Kenya, EMEP/IPTp-MON-Kenya, Sek-PNG, ECHO-DRC.

	FSP/ MISAME- BF (*N* = 1020)	STOPPAM- Benin (*N* = 791)	STOPPAM-Tanzania (*N* = 789)	ITN- Kenya (*N* = 711)	EMEP/IPTp-MON-Kenya (*N* = 471)	Sek-PNG (*N* = 293)	ECHO- DRC (*N* = 164)
Study enrollment (years)	2006–2008	2008–2010	2008–2010	1996–1999	2011–2013	2005–2007	2005–2006
Maternal age	23 (19.5–28)	25 (22–30)	26 (22–31)	24 (20–30)	24 (20–30)	24 (21–28)	27 (23.5–31)
Gravidity							
1 (Primi-)	205 (20)	147 (19)	162 (21)	127 (18)	94 (20)	115 (39)	43 (26)
2 (Secundi-)	216 (21)	173 (22)	201 (25)	118 (17)	77 (16)	54 (18)	22 (13)
3+ (Multi-)	599 (59)	471 (59)	426 (54)	466 (66)	300 (64)	124 (42)	99 (60)
Trimester*							
1	385 (38)	174 (22)	88 (11)	3 (0)	67 (14)	0 (0)	6 (4)
2	595 (58)	616 (78)	701 (89)	376 (53)	247 (52)	214 (73)	158 (96)
3	40 (4)	1 (0)	0 (0)	292 (41)	140 (30)	75 (26)	0 (0)
Missing GA	0 (0)	0 (0)	0 (0)	40 (6)	17 (4)	4 (1)	0 (0)
Anemic^†^							
Yes	372 (36)	354 (45)	289 (37)	416 (59)	169 (36)	272 (93)	43 (26)
No	630 (62)	433 (55)	497 (63)	293 (41)	297 (63)	21 (7)	107 (65)
Missing	18 (2)	4 (1)	3 (0)	2 (0)	5 (1)	0 (0)	14 (9)
HIV							
Yes	–	13 (2)	39 (4)	51 (7)	0 (0)	–	4 (2)
No	–	699 (88)	693 (88)	234 (33)	468 (99)	–	160 (98)
Missing	1,020 (100)	79 (10)	57 (7)	426 (60)	3 (1)	293 (100)	0 (0)
Area of Residence							
Rural	1,020 (100)	791 (100)	430 (55)	711 (100)	471 (100)	282 (96)	0 (0)
Urban	0 (0)	0 (0)	354 (45)	0 (0)	0 (0)	8 (3)	164 (100)
Missing	0 (0)	0 (0)	5 (1)	0 (0)	0 (0)	3 (1)	0 (0)
IPTp dosage	2 (1–2)	2 (2–2)	2 (2–2)	0 (0–0)	3 (2–3)	–	2 (2–2)
Bed net ownership							
Yes	–	254 (32)	571 (72)	348 (49)	–	240 (82)	164 (100)
No	–	537 (68)	52 (7)	363 (51)	–	49 (17)	0 (0)
Missing	1,020 (100)	0 (0)	166 (21)	0 (0)	471 (100)	4 (1)	0 (0)

Categorical variables are expressed as *N* (%) and continuous variables are expressed as median (IQR). A dash indicates information on particular factor was not assessed in parent study.

* Based on ultrasound if measured, otherwise based on Ballard’s score or symphysis-pubis fundal height (SFH). When using SFH, to adjust for misclassification in the first trimester, a fundal height <7 cm was defined as first trimester, while SFH < 28 cm was defined as second trimester, and SFH ≥ 28 cm was defined as third trimester.

^†^ Anemic = hemoglobin <11 g/dL of venous blood, if available, or hematocrit <33% in the first and third trimesters and less than 10.5 g/dL and 32%, respectively, for the second trimester.

BF, Burkina Faso; DRC, Democratic Republic of the Congo; EMEP, Evaluation of Medications used in Early Pregnancy; GA, gestational age; IPTp, intermittent preventive treatment in pregnancy; ITN, insecticide-treated bed nets; M3, Maternal Malaria and Malnutrition; PNG, Papua New Guinea; STOPPAM, Strategies to Prevent Pregnancy-Associated Malaria.

Five of the thirteen included studies were judged to be at a lower risk of bias for the assessment of interaction between malaria infection and maternal malnutrition on BW (S4 Table). Among the 8 other studies, 3 had a <75% retention rate for the primary outcome, 5 did not measure at least 80% of BWs with an electronic scale with known precision ≤20 g, and 3 did not measure at least 80% of BWs within 24 hours.

### Independent effects of malaria infection and malnutrition

**Fig 3:**
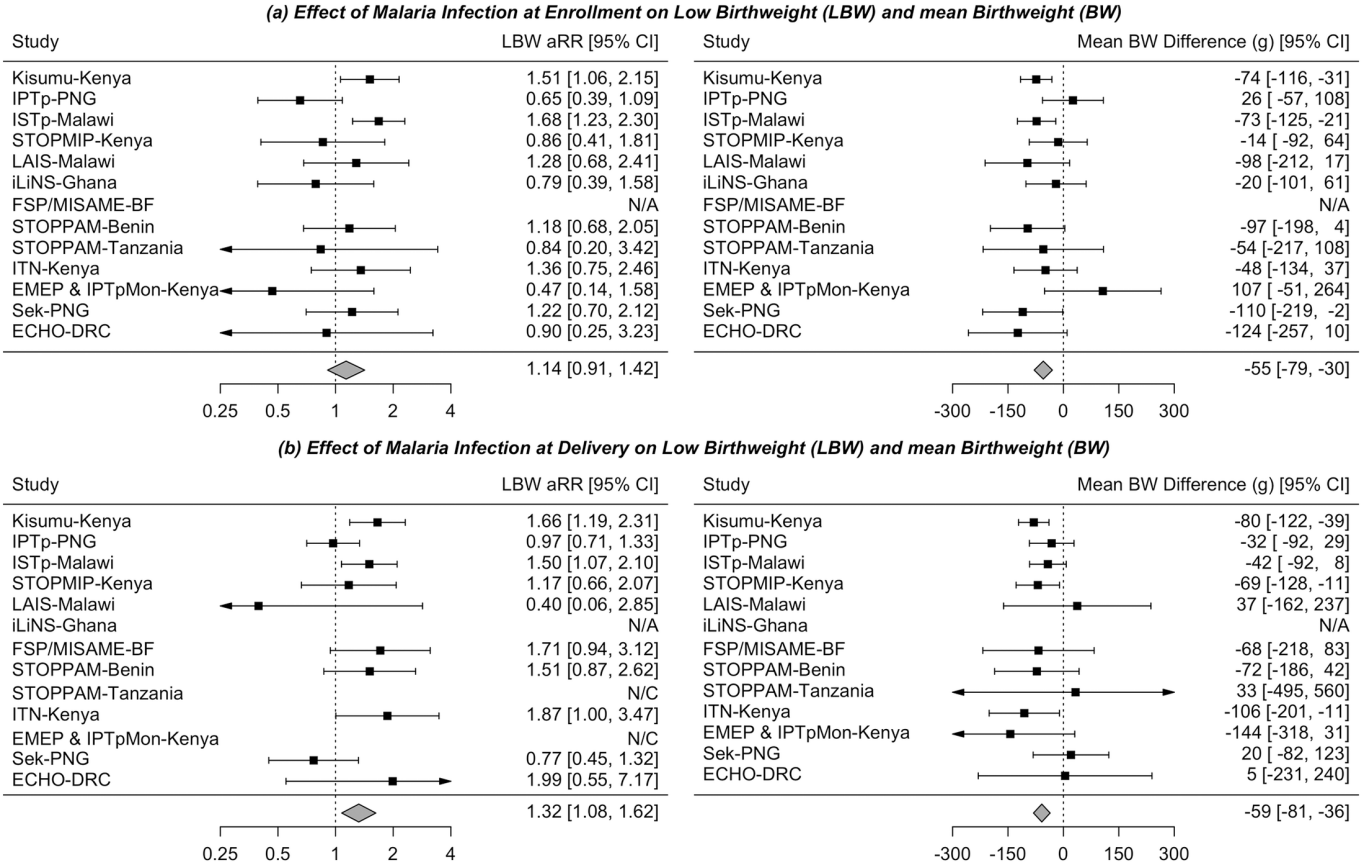
The independent effects of (a) malaria infection at enrollment and (b) malaria infection at delivery on risk of low birthweight and mean birthweight among women enrolled in 1 of 13 studies from the Maternal Malaria and Malnutrition (M3) initiative. The inverse probability of treatment weighted (IPTW) estimates controlled for confounding between malaria at enrollment and low birthweight (LBW) by maternal age, gravidity, area of residence, mid-upper arm circumference at enrollment, and HIV infection. IPTW estimates controlled for confounding between malaria at delivery and LBW and additionally controlled for anemia and number of doses of antimalarial intermittent preventive therapy received during pregnancy. aRR, adjusted risk ratio; BF, Burkina Faso; BW, Birthweight; CI, Confidence interval; DRC, Democratic Republic of the Congo; LBW, Low birthweight; N/A, not available; N/C, no model convergence; PNG, Papua New Guinea.

The pooled IPTW-adjusted risk ratio (aRR) for the effect of malaria infection at enrollment on LBW was 1.14 (95% CI: 0.91, 1.42; 95% *τ*^2^ = 0.05 [95% CI: 0.00, 0.25]; PEI: 0.72, 1.80), and the mean BW difference was −55 g (95% CI: −79, −30; *τ*^2^ = 0 [95% CI: 0.00, 1,610]) (Fig 3a). The effect of malaria infection at delivery was more pronounced: aRR, 1.32 (95% CI: 1.08, 1.62; *τ*^2^ = 0.04 [95% CI: 0.00, 0.39]; 95% PEI: 0.91, 1.91) (Fig 3b). When considering SGA and PTB as secondary outcomes, results were similar for malaria infection at enrollment and attenuated for malaria infection at delivery (S5 Table). The effect of malaria infection at enrollment was attenuated among those with more than 1 IPTp dose versus 1 or 0 doses (aRR 0.98 versus 1.22) and was slightly stronger among primi/secundigravid versus multigravida women (aRR 1.19 versus 1.14). A slightly stronger effect of malaria infection was seen among women enrolled in studies conducted prior to 2008, in Africa, or with malaria infection prevalence at or above the median (S2 Fig).

**Fig 4:**
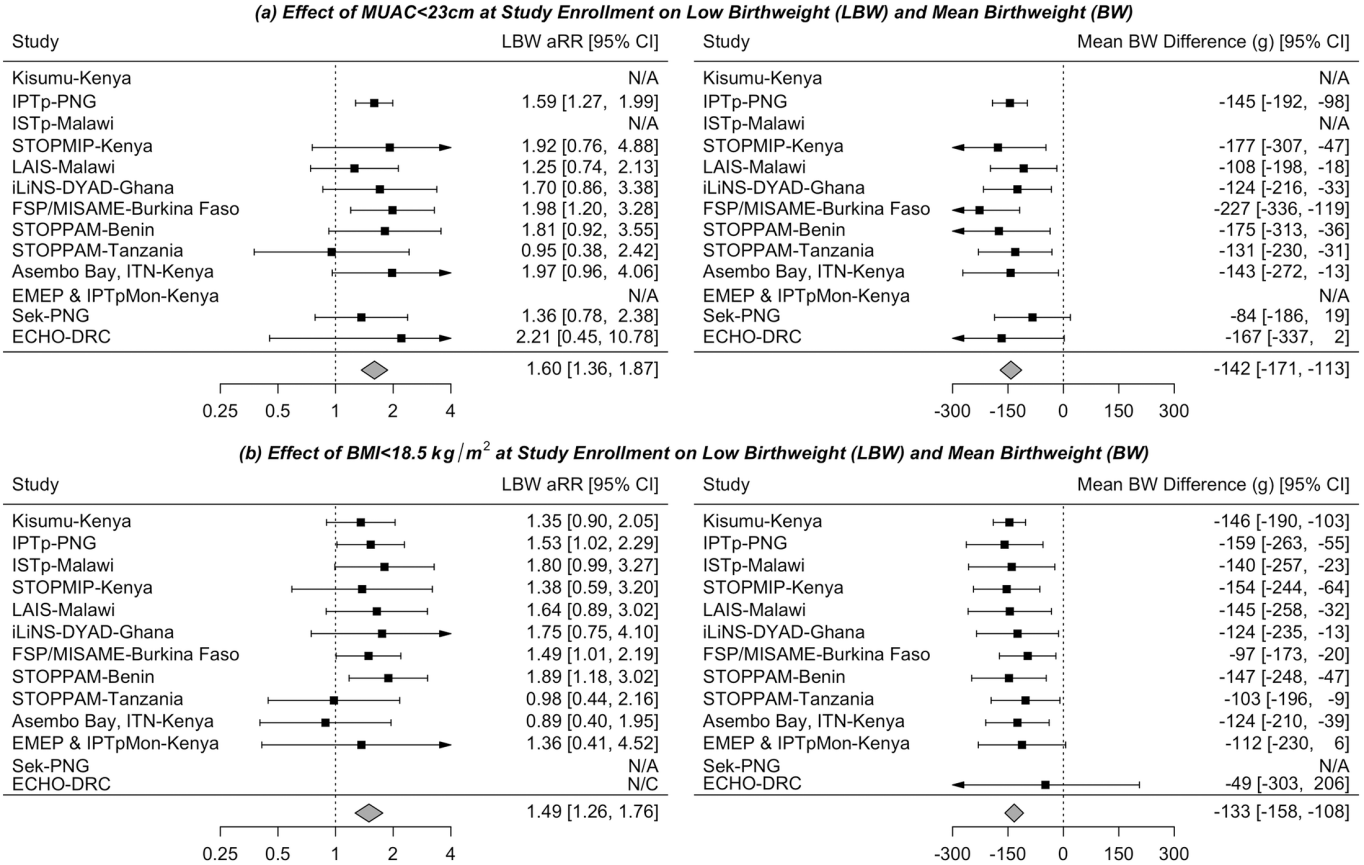
The independent effect of malnutrition at enrollment, (a) mid-upper arm circumference (MUAC) < 23 cm and (b) Body Mass Index (BMI)* < 18.5 kg/m^2^, on risk of low birthweight (LBW) and mean birthweight among 14,633 women enrolled in 1 of 13 studies from the Maternal Malaria and Malnutrition (M3) initiative from 1996 to 2015. BMI is adjusted for gestational age to reflect the estimated first trimester weight. Inverse probability of treatment weighted (IPTW) estimates controlling for confounding between malnutrition (MUAC or BMI) at enrollment and LBW by maternal age, gravidity, area of residence, anemia, and HIV infection (where available). BMI, Body mass index; BW, Birthweight. CI, Confidence interval; DRC, Democratic Republic of the Congo; MUAC, mid-upper arm circumference; N/A, not available; N/C, no model convergence; PNG, Papua New Guinea.

The aRR for the effect of low MUAC on LBW was 1.60 (95% CI: 1.36, 1.87; *τ*^2^ = 0 [95% CI: 0.00, 0.05]); the mean BW difference was −142 g (95% CI: −171, −113; *τ*^2^ = 0 [95% CI: 0, 100] (Fig 4a). Results were similar for low BMI: aRR, 1.49 (95% CI: 1.26, 1.76; *τ*^2^ = 0 [95% CI: 0.00, 0.16]); mean BW difference −133 g (95% CI: −158, −108; *τ*^2^ = 0 [95% CI: 0.00, 0.00]) (Fig 4b). There was no modification by study characteristics on the malnutrition–LBW relationship (S3 Fig). Similar but weaker trends were observed when SGA was used as the outcome among the studies with ultrasound data, but low MUAC or low BMI were significantly associated with an increased risk of PTB (S5 Table).

### Interaction and EMM

The joint aRR for both malaria infection at enrollment and low MUAC was 2.13 (95% CI: 1.21, 3.73; *τ*^2^ = 0.25 [95% CI: 0.00, 1.82]; 95% PEI: 0.80, 5.67), and the mean BW difference was −163 g (95% CI: −253, −75; *τ*^2^ = 6,995 [95% CI: 0, 58,414]; 95% PEI: −328, 0). The multiplicative interaction term for LBW was 1.30 (95% CI: 0.62, 2.72; *τ*^2^ = 0.37 [95% CI: 0.00, 3.97]; 95% PEI: 0.39, 4.31), the additive interaction term for LBW was −0.01 (95% CI: −0.09, 0.08; *τ*^2^ = 0.003 [95% CI: 0.00, 0.04]; 95% PEI: −0.11, 0.09), and the additive interaction term for mean BW difference was 38 g (95% CI: −90, 166; *τ*^2^ = 17,198 [95% CI: 0, 120,165]; 95% PEI: −219, 295). Sensitivity analyses that varied the definitions of malaria, malnutrition, outcome, and analytic approach largely did not qualitatively alter the results; however, restriction to adolescent women did suggest potential multiplicative and additive interaction between low MUAC and malaria infection at enrollment among this subgroup (product term 2.49 [95% CI: 0.88, 7.02]; additive interaction term 0.08 [95% CI: −0.07, 0.22]) (S6 Table). Additionally, meta-regression indicated apparent multiplicative interaction and slight additive interaction between MUAC and malaria infection at enrollment among studies conducted in Africa (multiplicative interaction term, 2.47 [95% CI: 1.12, 5.42]; additive interaction contrast, 0.06 [95% CI: −0.05, 0.17] S4 Fig), but this interaction was not seen when assessing malaria infection at delivery or BMI or when accounting for multiple comparisons with a Bonferroni correction (99% CI: 0.88, 6.95). In EMM analyses, the aRR for the effect of malaria infection at enrollment on LBW among low MUAC women was 1.32 (95% CI: 0.66, 2.63; *τ*^2^ = 0.43 [95% CI: 0.00, 3.40]; 95% PEI: 0.36, 4.79), compared to 0.98 (95% CI: 0.74, 1.29; *τ*^2^ = 0 [95% CI: 0.00, 0.32]) among well-nourished women.

The joint aRR for both malaria infection at delivery and low MUAC was 2.16 (95% CI: 1.25, 3.74; *τ*^2^ = 0.23 [95% CI: 0.00, 1.61]; 95% PEI: 0.84, 5.55), and the mean BW difference was −196 g (95% CI: −301, −92; *τ*^2^ = 10,904 [95% CI: 0, 86,721]; 95% PEI: −401, 8). The multiplicative interaction term for LBW was 0.82 (95% CI: 0.50, 1.33; *τ*^2^ = 0 [95% CI: 0.00, 3.79]), the additive interaction term for LBW was −0.01 (95% CI: −0.10, 0.07; *τ*^2^ = 0 [95% CI: 0.00, 0.06]), and the additive interaction term for mean BW difference was −49 g (95% CI: −190, 93; *τ*^2^ = 20,087 [95% CI: 0, 154,675]; 95% PEI: −326, 229).

## Discussion

Using the large M3 initiative dataset, we found that pregnant women who were both infected with malaria and malnourished were at greater risk of LBW and reduced mean BW compared to their uninfected, well-nourished counterparts, but there was overall no convincing evidence of synergism, i.e., excess risk due to interaction. This finding was consistent for both time points of malaria diagnosis (at enrollment and delivery) and both definitions of malnutrition (MUAC and BMI). This suggests that malaria infection and malnutrition largely act independently to influence fetal growth and gestational length.

A 2004 review estimated that women infected with placental malaria were twice as likely to have a LBW infant [51]. Our findings are broadly consistent with this review, although with weaker effects on LBW (overall aRR for malaria infection at delivery: 1.32 [95% CI: 1.08, 1.62], aRR restricted to African studies: 1.55 [95% CI: 1.29, 1.85]), possibly reflecting increased access to preventive strategies and fewer chronic infections [3,4]. In support of this hypothesis, the effect of malaria infection on LBW appears lower in women who received more doses of IPTp. The effects of malaria infection at enrollment on LBW were weaker than at delivery, contradicting the theory that malaria infection earlier in pregnancy is more disruptive to placental function [2]. This weaker effect at enrollment could either suggest that antimalarial treatment, provided in most studies, cleared infection and allowed catch-up growth or that infection at delivery represents more severe infections that were not cleared despite medications. Both malaria infection at enrollment and delivery were associated with a reduction in BW of around 55 grams, which has been found in other studies [52].

Our data are consistent with a 2011 meta-analysis, which estimated that underweight women had increased risk of LBW (aRR: 1.64 [95% CI: 1.38, 1.94]), although studies included in that meta-analysis used different definitions for underweight [53]. In our study, using consistent cutoffs of malnutrition across studies, both low MUAC (aRR 1.60 [95% CI: 1.36, 1.87]) and low BMI (aRR 1.49 [95% CI: 1.26, 1.76]) increased the risk of LBW. This information is consistent with other evidence that adequate maternal nutrition is integral for fetal growth [5].

Prior literature on the interaction between malaria infection and malnutrition is sparse. Two studies in the DRC and Kenya showed that the association between malaria infection and reduced fetal growth was greatest among malnourished women [10,11]. In a third study in Benin, the effect of malaria infection on fetal growth velocity was greatest among women with low anthropometric status, but there was no modification by maternal nutrition on the effect of malaria infection on BW z-scores. A fourth study in PNG found that the effect of histology-defined placental malaria infection on LBW was higher among women with a low BMI, but that study found that malnutrition did not modify the association between peripheral blood malaria infection parasitemia and SGA [9]. The Benin, Congo, and PNG studies were included in the present analysis, but our analytic approach differed from the original publications in the assessment of both interaction and modification. Unlike these prior studies, our pooled results suggest that there is a negligible impact of maternal anthropometry on the relationship between malaria infection and LBW and further indicate that there is no evidence of excess risk of LBW due to interaction (i.e., synergism). There was some indication of multiplicative and additive interaction between low MUAC and malaria infection at enrollment among adolescent women; however, these estimates were very imprecise and were only pooled across 4 studies that enrolled enough adolescent women to assess this subgroup. Adolescent women are recognized to be at high risk of adverse pregnancy outcomes [54], and tailored antenatal care programs addressing malaria, nutrition, and other health issues should be considered for this group. In an a priori sensitivity analysis restricted to African studies, there was apparent interaction between malaria infection at enrollment and MUAC, which is consistent with the prior publications. Regional differences could be due to genetics, low MUAC, or anemia prevalence; however, these subregion effects were not statistically significant when properly accounting for multiple comparisons and were absent when using other definitions of malaria (i.e., at delivery) or malnutrition (i.e., BMI). Additionally, the additive interaction, which has been argued to be the more relevant measure for public health impact [55], was only slightly elevated among the African studies. Notably, only 183 women (2%) were jointly infected and malnourished (low MUAC). Thus, even if there is a multiplicative interaction between malaria infection and MUAC among African women or among adolescent women, the proportion of women implicated is small, and does not indicate a large public health burden. However, even in the absence of strong interaction between malaria infection and malnutrition on LBW, we emphasize that interventions on both malaria infection and malnutrition are warranted given their independent effects.

This work had several strengths and limitations. We substantially increased the number of women in whom the hypothesized interaction between malaria infection, malnutrition, and LBW was investigated; notably, the number of pregnant women from Africa was almost 10 times more than all prior studies. Analyzed studies were performed in a variety of settings, increasing the generalizability of these results. Furthermore, availability of individual-level data enabled us to harmonize definitions and minimize heterogeneity. Our work is strengthened by providing results for SGA and PTB as secondary outcomes, which showed findings consistent with LBW. However, we were only able to assess SGA and PTB among a subset of nine of the 13 studies with available ultrasound-dated gestational age. There is no alternative satisfactory dating tool to ultrasound in later pregnancy, thus we used all ultrasound data provided regardless of gestation. Some women were enrolled after 24 weeks gestation (S2 Table), reducing the accuracy of ultrasound among these pregnancies and potentially underestimating gestational age in some SGA babies. Missing data were imputed using multivariate normal multiple imputation, and while not all variables followed a normal distribution (e.g., the binary variable LBW), simulation studies have shown that multivariate normal multiple imputation provides less biased estimates than complete-case analysis even when imputing binary or ordinal variables [56]. We were obliged to pool malaria diagnostics of varying sensitivity and specificity, and we were limited to 2 cross-sectional assessments of malaria infection. Nevertheless, sensitivity analyses that evaluated alternative definitions of malaria, or incorporated repeat diagnostics during pregnancy, were consistent with the main results. Additionally, there may be selection bias due to excluding pregnancy losses. There were only 116 (3%) pregnancy losses in 4 studies (*N* = 4,571) in the M3 initiative that collected these data, but this is almost certainly an underestimate, since many studies enrolled women after the first trimester. We were obliged to extrapolate prepregnancy BMI using gestational age and BMI at enrollment. Additionally, the M3 initiative represents a convenience sample of available and eligible studies identified through the MiPc and not an exhaustive aggregation of all existing studies available to assess interactions between malaria and malnutrition on LBW. This could potentially lead to selection bias if selection of studies were associated with the effect estimates in that study; however, we did not observe any qualitative differences between studies providing individual participant data and those studies not included in the meta-analysis (S3 Table). Furthermore, women enrolled in studies were likely healthier and received better antenatal care than the general population; the effects of malaria and malnutrition in reality might well be greater than were observed within these research settings. The risk of bias assessment identified 10 studies as being at a higher risk of bias, primarily due to BW not being measured with an electronic scale within 24 hours of delivery. Finally, we cannot discount possible unmeasured confounding, particularly by helminth infections, sexually transmitted infections, environmental pollutants, or micronutrient deficiencies; however, it is important to note that because neither malnutrition nor malaria could be randomized, large-scale, multisite cohort analyses such as this one are necessarily the gold standard for addressing these scientific questions. Future studies may wish to assess joint effects of malaria with other nutritional indicators (e.g., height, obesity, anemia, other micronutrients). Additionally, future studies may wish to further investigate possible interactions between malaria infection and malnutrition on risk of LBW in adolescent mothers.

In summary, our findings suggest that women who are both infected with malaria and malnourished are at greater risk of LBW than their uninfected, well-nourished counterparts but that there is no conclusive evidence of synergistic interaction between the 2. Rather, we propose that malaria infection and malnutrition act independently to disrupt fetal growth and that malnutrition in particular has a strong effect on LBW. Of all 14,633 pregnancies, 35% were affected by malaria infection and/or malnutrition, illustrating the high burden of at-risk pregnancies in LMICs. Malaria infection and malnutrition represent 2 established and modifiable causes of LBW that should both be addressed to optimize pregnancy outcomes in LMIC.

## Supporting information

S1 Table Characteristics of the 13 individual studies included in the Maternal Malnutrition and Malaria (M3) initiative. (DOCX)

S2 Table Descriptions of the scales used to measure birthweight, how gestational age was assessed, and the median gestational age for each of the 13 studies in the Maternal Malaria and Malnutrition (M3) initiative. (DOCX)

S3 Table Characteristics of studies not included in the Maternal Malaria and Malnutrition (M3) initiative cohort. (DOCX)

S4 Table Assessment of risk of bias for the 13 studies included in the individual participant data meta-analysis. (DOCX)

S5 Table The independent and joint effects of malaria infection at enrollment, malaria infection at delivery, low mid-upper arm circumference (MUAC), and low body mass index (BMI) on the risk of small for gestational age (SGA) and risk of preterm birth among a subset of 9 studies from the Maternal Malaria and Malnutrition (M3) initiative. (DOCX)

S6 Table Select sensitivity analysis results for the multiplicative interaction effects for malaria and malnutrition on risk of adverse birth outcomes among the 13 studies in the Maternal Malaria and Malnutrition (M3) initiative. Sensitivity analyses varied the definitions of malaria, malnutrition, the outcome of interest, and the approach taken in pooling study results.
(DOCX)

S7 Table PRISMA 2009 checklist. (DOC)

S8 Table Individual participant data checklist. (DOCX)

S1 Text Multiple imputation. (DOCX)

S2 Text Protocol for the individual participant data project. Written 17 November 2014.
(DOCX)

S1 Fig Prevalence of low mid-upper arm circumference (MUAC < 23cm) compared to prevalence of low body mass index (BMI < 18.5 kg/m^2^) among the 13 studies in the Maternal Malaria and Malnutrition (M3) initiative. (DOCX)

S2 Fig Meta-regression results for the effects of malaria infection at enrollment and delivery on risk of low birthweight (LBW) and mean birthweight (BW) by time period, study type, location, and malaria prevalence. Median malaria prevalence across studies was 17% at enrollment and 15% at delivery. RCT = randomized control trial.
(DOCX)

S3 Fig Meta-regression results for the effects of malnutrition at enrollment, (a) low mid-upper arm circumference (MUAC < 23 cm) and (b) low BMI (BMI < 18.5 kg/m^2^), on risk of low birthweight (LBW) and mean birthweight (BW) by time period, study type, location, and malaria prevalence. Median malaria prevalence across studies was 17% at enrollment and 15% at delivery. RCT = randomized control trial.
(DOCX)

S4 Fig Meta-regression results for the multiplicative and additive interaction effects for malaria at enrollment or delivery and low mid-upper arm circumference (MUAC < 23 cm) on risk of low birthweight (LBW) and mean birthweight (BW) by time period, study type, location, and malaria prevalence. Median malaria prevalence across studies was 17% at enrollment and 15% at delivery.
(DOCX)

S5 Fig Prevalence of malaria infection at delivery among the 13 studies in the Maternal Malaria and Malnutrition (M3) initiative. (DOCX)

## Abbreviations

aRR: adjusted risk ratio
BMI: body mass index
BW: birthweight
CI: confidence interval
DRC: Democratic Republic of the Congo
EMM: effect measure modification
FGR: fetal growth restriction
HIV: human immunodeficiency virus
IPTp: intermittent preventive treatment in pregnancy
IPTW: inverse probability of treatment weights
LBW: low birthweight
LM: light microscopy
LMIC: low- and middle-income countries
M3: Maternal Malaria and Malnutrition
MiPc: Malaria in Pregnancy Consortium
MUAC: mid-upper arm circumference
PCR: polymerase chain reaction
PEI: population effects interval
PNG: Papua New Guinea
RDT: rapid diagnostic test
RR: risk ratio
SGA: small for gestational age
SP: sulfadoxine-pyrimethamine
WHO: World Health Organization
