# Supplementary material for: Malaria, malnutrition, and birthweight: A meta-analysis using individual participant data
Source: PLoS Med. 2017 Aug 8;14(8):e1002373. doi: 10.1371/journal.pmed.1002373 (PMC5549702; doi:10.1371/journal.pmed.1002373)
Supplement: S1 Table — (DOCX) [file pmed.1002373.s001.docx]

| Countries | Study Name | Design | Period | Median GA (IQR)* | Sample size included in M3 |
| --- | --- | --- | --- | --- | --- |
| Kenya | Kisumu cohort | Cohort | 1996-2001 | 36 (34-37) | 3388** |
| PNG | IPTp study | RCT | 2009-2013 | 22 (19-25) | 1943 |
| Malawi | ISTp | RCT | 2011-2013 | 21 (19-23) | 1602 |
| Kenya | STOPMIP | RCT | 2012-2015 | 23 (20-26) | 1203 |
| Malawi | LAIS | RCT | 2003-2006 | 20 (18-23) | 1190 |
| Ghana | iLiNS-DYAD | RCT | 2009-2012 | 17 (15-20) | 1068 |
| BF | FSP/MISAME | RCT | 2006-2008 | 16 (11-21) | 1020 |
| Benin | STOPPAM I | Cohort | 2008-2010 | 17 (14-20) | 791 |
| Tanzania | STOPPAM II | Cohort | 2008-2010 | 19 (15-21) | 789 |
| Kenya | ITN | RCT | 1996-1999 | 24 (20-30) | 711 |
| Kenya | EMEP & IPTpMon | Cohort ^#^ | 2011-2013 | 23 (16-29) | 471^#^ |
| PNG | Sek cohort | Cohort | 2005-2007 | 25 (22-28) | 293 |
| DRC | ECHO | Cohort | 2005-2006 | 19 (17-21) | 164 |

BF=Burkina Faso. DRC=Democratic Republic of the Congo. IPT (doses)=intermittent preventive treatment in pregnancy. IST=intermittent screening for malaria infection. PNG=Papua New Guinea. RCT=randomised controlled trial. SP=sulphadoxine-pyrimethamine.

* Median (IQR): Gestational age at enrolment assessed by fetal biometry, or symphysis-pubis fundal height when ultrasound unavailable

# The EMEP study was a prospective cohort study with some overlapping enrolment with the cross-sectional study IPTp-MON. 111 pregnancies were enrolled in both EMEP and IPTp- MON; information on malaria infection at delivery was obtained for the subset of women in IPTp-MON.

**Includes additional women from a sub-study not included in the parent study which otherwise met inclusion criteria for the pooled data.
