# Supplementary material for: Malaria, malnutrition, and birthweight: A meta-analysis using individual participant data
Source: PLoS Med. 2017 Aug 8;14(8):e1002373. doi: 10.1371/journal.pmed.1002373 (PMC5549702; doi:10.1371/journal.pmed.1002373)
Supplement: S2 Table — (DOCX) [file pmed.1002373.s002.docx]

| **Countries** | **Study Name** | **Birth Weight Scale** | **Birth Weight Precision** | **Gestational Age Assessment** | **Median gestational age at enrollment (IQR)** |
| --- | --- | --- | --- | --- | --- |
| Kenya | Kisumu cohort | Electronic balance | 10 g | SFH | 36 (34-37) |
| PNG | IPTp study | Digital infant scale | 10 g | Ultrasound | 22 (19-25) |
| Malawi | ISTp | Digital scale | 10 g | Ultrasound | 21 (19-23) |
| Kenya | STOPMIP | Digital scale | 10 g | SFH, Ballard Score | 23 (20-26) |
| Malawi | LAIS | Spring scale or electronic infant weighing scale | 50 g (if spring); 10 g (if digital) | Ultrasound | 20 (18-23) |
| Ghana | iLiNS-DYAD | SECA 383 | 20 g | Ultrasound | 17 (15-20) |
| Burkina Faso | FSP/MISAME | SECA 813 | 100 g | Ultrasound | 16 (11-21) |
| Benin | STOPPAM I | Electronic scale Seca | 2 g | Ultrasound | 17 (14-20) |
| Tanzania | STOPPAM II | Spring scale or a digital strain gauge scale | 50 g (if spring); 10 g (if digital) | Ultrasound | 19 (15-21) |
| Kenya | ITN | Hanging weighing scale | 10 g | SFH | 24 (20-30) |
| Kenya | EMEP & IPTpMon | Digital scales | 10 g | Ultrasound, SFH | 23 (16-30) |
| PNG | Sek cohort | SECA baby scale | 10 g | SFH | 25 (22-28) |
| DRC | ECHO | SECA digital scale Model 890 | 1 g | Ultrasound | 19 (17-21) |

DRC=Democratic Republic of the Congo. IQR=interquartile range. SFH=Symphysis-pubis fundal height. PNG=Papua New Guinea.
