# Supplementary material for: Malaria, malnutrition, and birthweight: A meta-analysis using individual participant data
Source: PLoS Med. 2017 Aug 8;14(8):e1002373. doi: 10.1371/journal.pmed.1002373 (PMC5549702; doi:10.1371/journal.pmed.1002373)
Supplement: S3 Table — (DOCX) [file pmed.1002373.s003.docx]

| **Countries** | **Study Name** | **Design** | **Period** | **Gestational Age at enrollment** | ***N*^‡^** | **Reason not included in IPD Meta-Analysis** | **Reference** |
| --- | --- | --- | --- | --- | --- | --- | --- |
| **Assessed for eligibility into the M3 cohort.** | | | | | | | |
| Malawi | iLiNS-Dyad-Ghana | RCT | 2011-2013 | <20 weeks | 1391 | Data were not yet available for inclusion | Ashorn 2015 [1] |
| Kenya | No specific name | Cohort | 2006-2009 | 18-35 weeks | 477 | Recruited women comparatively late in pregnancy | McClure 2014 [2] |
| Malawi | APPLe trial | RCT | 2004-2007 | <24 weeks | 2,149 | Did not directly measured the number of SP doses given for IPTp. | van den Broek 2014 [3] |
| Benin, Gabon, Mozambique, Tanzania | MiPPAD- HIV-uninfected | RCT | 2009-2013 | ≤28 weeks | 4,749 | Did not assess malaria at antenatal enrolment | González 2014 [4] |
| Kenya, Mozambique, Tanzania | MiPPAD- HIV-infected | RCT | 2010-2013 | ≤28 weeks | 1,071 | Did not assess malaria at antenatal enrolment | González 2014 [5] |
| **Identified after compilation of M3 dataset.** | | | | | | | |
| Kenya | Iron supplementation and malaria study | RCT | 2011-2013 | 13-23 weeks | 470 | Identified after compilation of M3 dataset | Mwangi 2015 [6] |
| Uganda | PROMOTE-BC1 | RCT | 2014 | 12-20 weeks | 300 | Identified after compilation of M3 dataset | Kakuru 2016 [7] |
| Benin | Anaemia in Pregnancy: Etiologies and Consequences “APEC” | Cohort | 2010-2011 | ≤28 weeks | 1,005 | Identified after compilation of M3 dataset | Ouédraogo 2013 [8] |
| Kenya | Chulaimbo Sub-District Hospital study |  | 2011-2012 | ≤26 weeks | 54 | Identified after compilation of M3 dataset | Toko 2016 [9] |
| Benin | Ouidah study | Cohort and trial | 2004-2006 | 16-28 weeks | 1,699 | Did not assess malaria at enrollment | Briand 2008 [10] |

HIV=Human immunodeficiency virus. IPD=Individual participant data. RCT=randomized controlled trial.

**REFERENCES:**

1. Ashorn P, Alho L, Ashorn U, Cheung YB, Dewey KG, Harjunmaa U, et al. The impact of lipid-based nutrient supplement provision to pregnant women on newborn size in rural Malawi: a randomized controlled trial. Am J Clin Nutr. 2015 Feb 1;101(2):387–97.

2. McClure EM, Meshnick SR, Lazebnik N, Mungai P, King CL, Hudgens M, et al. A cohort study of Plasmodium falciparum malaria in pregnancy and associations with uteroplacental blood flow and fetal anthropometrics in Kenya. Int J Gynaecol Obstet. 2014 Jul;126(1):78–82.

3. van den Broek NR, White SA, Goodall M, Ntonya C, Kayira E, Kafulafula G, et al. The APPLe Study: A Randomized, Community-Based, Placebo-Controlled Trial of Azithromycin for the Prevention of Preterm Birth, with Meta-Analysis. PLoS Med. 2009 Dec 1;6(12):e1000191.

4. González R, Mombo-Ngoma G, Ouédraogo S, Kakolwa MA, Abdulla S, Accrombessi M, et al. Intermittent Preventive Treatment of Malaria in Pregnancy with Mefloquine in HIV-Negative Women: A Multicentre Randomized Controlled Trial. PLoS Med. 2014 Sep 23;11(9):e1001733.

5. González R, Desai M, Macete E, Ouma P, Kakolwa MA, Abdulla S, et al. Intermittent Preventive Treatment of Malaria in Pregnancy with Mefloquine in HIV-Infected Women Receiving Cotrimoxazole Prophylaxis: A Multicenter Randomized Placebo-Controlled Trial. PLOS Med. 2014 Sep 23;11(9):e1001735.

6. Mwangi MN, Roth JM, Smit MR, Trijsburg L, Mwangi AM, Demir AY, et al. Effect of Daily Antenatal Iron Supplementation on Plasmodium Infection in Kenyan Women: A Randomized Clinical Trial. JAMA. 2015 Sep 8;314(10):1009–20.

7. Kakuru A, Jagannathan P, Muhindo MK, Natureeba P, Awori P, Nakalembe M, et al. Dihydroartemisinin-Piperaquine for the Prevention of Malaria in Pregnancy. N Engl J Med. 2016 Mar 10;374(10):928–39.

8. Ouédraogo S, Koura GK, Accrombessi MMK, Bodeau-Livinec F, Massougbodji A, Cot M. Maternal Anemia at First Antenatal Visit: Prevalence and Risk Factors in a Malaria-Endemic Area in Benin. Am J Trop Med Hyg. 2012 Sep 5;87(3):418–24.

9. Toko EN, Sumba OP, Daud II, Ogolla S, Majiwa M, Krisher JT, et al. Maternal Vitamin D Status and Adverse Birth Outcomes in Children from Rural Western Kenya. Nutrients. 2016 Dec 7;8(12).

10. Briand V, Denoeud L, Massougbodji A, Cot M. Efficacy of Intermittent Preventive Treatment versus Chloroquine Prophylaxis to Prevent Malaria during Pregnancy in Benin. J Infect Dis. 2008 Aug 15;198(4):594–601.
