# Supplementary material for: Malaria, malnutrition, and birthweight: A meta-analysis using individual participant data
Source: PLoS Med. 2017 Aug 8;14(8):e1002373. doi: 10.1371/journal.pmed.1002373 (PMC5549702; doi:10.1371/journal.pmed.1002373)
Supplement: S4 Table — (DOCX) [file pmed.1002373.s004.docx]

| **Countries** | **Study Name** | **≥75% Retention Rate for primary outcome** | **Measurement of Important Confounders*** | **Is it clearly described how parasitemia was measured?** | **Was MUAC and/or BMI measured?** | **>80% of birth weights measured using electronic scale with known precision ≤20g** | **>80% Birth weight measured within 24 hours** | **Lower risk of bias**^†^ |
| --- | --- | --- | --- | --- | --- | --- | --- | --- |
| Kenya | Kisumu cohort | Yes | Yes | Yes | Yes | Yes | Yes | Yes |
| PNG | IPTp study | No | Yes | Yes | Yes | Yes | Yes | No |
| Malawi | ISTp | Yes | Yes | Yes | Yes | Yes | Yes | Yes |
| Kenya | STOPMIP | Yes | Yes | Yes | Yes | Yes | Yes | Yes |
| Malawi | LAIS | Yes | Yes | Yes | Yes | No | Yes | No |
| Ghana | iLiNS-DYAD | Yes | Yes | Yes | Yes | Yes | No | No |
| Burkina Faso | FSP/MISAME | Yes | Yes | Yes | Yes | No | Yes | No |
| Benin | STOPPAM I | Yes | Yes | Yes | Yes | Yes | Yes | Yes |
| Tanzania | STOPPAM II | Yes | Yes | Yes | Yes | No | Yes | No |
| Kenya | ITN | Yes | Yes | Yes | Yes | No | Yes | No |
| Kenya | EMEP & IPTpMon | No | Yes | Yes | Yes | Yes | No | No |
| PNG | Sek cohort | No | Yes | Yes | Yes | No | No | No |
| DRC | ECHO | Yes | Yes | Yes | Yes | Yes | Yes | Yes |

***** Important confounders include maternal age, gravidity, rural versus urban residence, HIV infection, and anemia at enrollment.

† Lower risk of bias based on “yes” to all: 75% Retention Rate, measurement of important confounders, is it clearly described how parasitemia and MUAC/BMI were measured,, electronic scale used for birth weight measurements with known precision≤20g, and birth weight measured within 24 hours.
