# Supplementary material for: Malaria, malnutrition, and birthweight: A meta-analysis using individual participant data
Source: PLoS Med. 2017 Aug 8;14(8):e1002373. doi: 10.1371/journal.pmed.1002373 (PMC5549702; doi:10.1371/journal.pmed.1002373)
Supplement: S5 Table — (DOCX) [file pmed.1002373.s005.docx]

|  |  | SGA | Preterm birth |
| --- | --- | --- | --- |
| Independent effects |  | aRR (95% CI) | |
| Malaria |  |  |  |
| Malaria infection at enrollment | | 1.16 (0.96, 1.41) | 1.15 (0.96, 1.38) |
| Malaria infection at delivery | | 1.09 (0.88, 1.35) | 1.17 (0.88, 1.56) |
| Malnutrition |  |  |  |
| MUAC<23 cm |  | 1.35 (1.10, 1.64) | 1.71 (1.14, 2.58) |
| BMI<18.5 kg/m^2^ |  | 1.17 (0.99, 1.38) | 1.48 (1.17, 1.87) |
| Joint exposure |  | Product term (95% CI)* | |
| Malaria timing | Malnutrition definition | |  |
| Enrollment | MUAC<23 cm | 0.94 (0.47, 1.85) | 1.19 (0.45, 3.16) |
| Enrollment | BMI<18.5 kg/m^2^ | 1.01 (0.46, 2.21) | 1.14 (0.54, 2.38) |
| Delivery | MUAC<23 cm | 1.72 (0.85, 3.48) | 0.40 (0.21, 0.74) |
| Delivery | BMI<18.5 kg/m^2^ | 0.82 (0.17, 3.85) | 0.86 (0.30, 2.45) |

BMI=body mass index. CI= confidence interval LBW=low birthweight. MUAC=mid-upper arm circumference. SGA=small-for-gestational age.

* Estimate of the departure from multiplicative interaction.
