# Supplementary material for: Malaria, malnutrition, and birthweight: A meta-analysis using individual participant data
Source: PLoS Med. 2017 Aug 8;14(8):e1002373. doi: 10.1371/journal.pmed.1002373 (PMC5549702; doi:10.1371/journal.pmed.1002373)
Supplement: S6 Table — Sensitivity analyses varied the definitions of malaria, malnutrition, the outcome of interest, and the approach taken in pooling study results. (DOCX) [file pmed.1002373.s006.docx]

| Malaria Definition | Malnutrition Definition | Method | Product term* (95% CI) |
| --- | --- | --- | --- |
| LBW |  |  |  |
| Enrollment | MUAC<23 cm | Two-stage | 1.30 (0.62, 2.72) |
| Enrollment | MUAC<23 cm | Two-stage**^†^** | 1.44 (0.64, 3.25) |
| Enrollment | MUAC<23 cm | Two-stage** | 2.49 (0.88, 7.02) |
| Enrollment | BMI<18.5 kg/m^2^ | Two-stage | 1.26 (0.77, 2.05) |
| Enrollment | MUAC<23 cm | One-stage | 1.12 (0.71, 1.78) |
| Delivery | MUAC<23 cm | Two-stage | 0.82 (0.50, 1.33) |
| Delivery | BMI<18.5 kg/m^2^ | Two-stage | 0.90 (0.56, 1.45) |
| Enrollment- with PCR | MUAC<23 cm | Two-stage | 1.57 (0.81, 3.05) |
| Delivery- with PCR | MUAC<23 cm | Two-stage | 0.80 (0.41, 1.56) |
| Any malaria^‡^ | MUAC<23 cm | Two-stage | 1.32 (0.71, 2.44) |
| Any malaria^‡^ | BMI<18.5 kg/m^2^ | Two-stage | 0.82 (0.54, 1.24) |

BMI=body mass index. BW=birth weight. CI= confidence interval LBW=low birthweight. MUAC=mid-upper arm circumference. PCR=polymerase-chain reaction. SGA=small-for-gestational age.

* Estimate of the departure from multiplicative interaction.

† Analysis included only birth weights measured within 24 hours of delivery.

‡Any malaria infection defined as a positive LM, RDT, or PCR at enrolment, delivery, or during pregnancy (among studies that were able to retrospectively share repeat diagnostics during pregnancy).

**Analysis restricted to adolescent women (ages ≤19).
