# Supplementary material for: Malaria, malnutrition, and birthweight: A meta-analysis using individual participant data
Source: PLoS Med. 2017 Aug 8;14(8):e1002373. doi: 10.1371/journal.pmed.1002373 (PMC5549702; doi:10.1371/journal.pmed.1002373)
Supplement: S8 Table — (DOCX) [file pmed.1002373.s008.docx]

| Item number |  | Reported in Paragraph # (per Section) |
| --- | --- | --- |
| 1 | Whether there was a protocol for the individual participant data project, and where it can be found | Included as S2 Text |
| 2 | Whether ethics approval was necessary and (if appropriate) granted | Methods, paragraph 1 |
| 3 | Why the individual participant data approach was initiated | Introduction, paragraphs 1-5 |
| 4 | The process used to identify relevant studies for the meta-analysis | Methods, paragraph 1 |
| 5 | How many authors (or collaborating groups) were approached for individual participant data, and the proportion that provided such data | Results, paragraph 1, Fig 1 |
| 6 | The number of authors who did not provide individual participant data, the reasons why, and the number of patients (and events) in the respective study | Results, paragraph 1; Supplemental Table 3 |
| 7 | Whether those authors who provided individual participant data gave all their data or only a proportion; if the latter, then describe what information was omitted and why | Methods, paragraph 1 |
| 8 | Whether there were any qualitative or quantitative differences between those studies providing individual participant data and those studies not providing individual participant data (if appropriate) | Discussion, paragraph 5; Supplemental Table 3 |
| 9 | The number of patients within each of the original studies and, if appropriate, the number of events | Table 1; Figure 1 |
| 10 | Details of any missing individual level data within the available individual participant data for each study, and how this was handled within the meta-analyses performed | Methods, paragraph 7; Table 1; Supplemental Text 1 |
| 11 | Details and reasons for including (or excluding) patients who were originally excluded (or included) by the source study investigators | Methods, paragraph 1 |
| 12 | Whether a one step or a two step individual participant data meta-analysis was performed, and the statistical details thereof, including how clustering of patients within studies was accounted for | Methods, paragraph 7 |
| 13 | How many patients from each study were used in each meta-analysis performed | Table 1; Figures 3-5 |
| 14 | Whether the assumptions of the statistical models were validated (for example, proportional hazards) within each study | Methods, paragraphs 7-8 |
| 15 | Whether the individual participant data results for each study were comparable with the published results, and, if not, why not (for example, individual participant data contained updated or modified information) | Discussion, paragraphs 1-4 |
| 16 | How individual participant data and non-individual participant data studies were analysed together (if appropriate). | N/A |
| 17 | The robustness of the meta-analysis results following the inclusion or exclusion of nonindividual participant data studies (if appropriate) | N/A |
