# Supplementary material for: Malaria, malnutrition, and birthweight: A meta-analysis using individual participant data
Source: PLoS Med. 2017 Aug 8;14(8):e1002373. doi: 10.1371/journal.pmed.1002373 (PMC5549702; doi:10.1371/journal.pmed.1002373)
Supplement: S1 Text — (DOCX) [file pmed.1002373.s009.docx]

Partially missing data were imputed using multiple imputation under the assumption of data missing at random (MAR). PROC MI SAS 9.3 was used to implement multiple imputation by data augmentation, which assumes data are multivariate normal. Variables included in the imputation models include variables in the final marginal structural model (malaria infection [at enrollment or delivery, analysis dependent], malnutrition (MUAC or BMI), interaction term for malaria and malnutrition, low birthweight, variables in the IPTW model (maternal age, gravidity, anemia, HIV, IPTp doses, bed net use at enrollment) and auxiliary variables to help improve the likelihood of the imputation model meeting the MAR assumption (gestational age at enrollment)[1,2]. A different imputation model was run separately for each study, with 40 imputations per study. PROC MIANALYZE was used to combine the effect estimates across imputations.

**References:**

1. Moons KGM, Donders RART, Stijnen T, Harrell FE. Using the outcome for imputation of missing predictor values was preferred. J Clin Epidemiol. 2006 Oct;59(10):1092–101.

2. Seaman SR, Bartlett JW, White IR. Multiple imputation of missing covariates with non-linear effects and interactions: an evaluation of statistical methods. BMC Med Res Methodol. 2012;12:46.
