# Supplementary material for: Malaria, malnutrition, and birthweight: A meta-analysis using individual participant data
Source: PLoS Med. 2017 Aug 8;14(8):e1002373. doi: 10.1371/journal.pmed.1002373 (PMC5549702; doi:10.1371/journal.pmed.1002373)
Supplement: S2 Text — Written 17 November 2014. (DOCX) [file pmed.1002373.s010.docx]

**Protocol for the individual participant data project: Malaria, Malnutrition, and Birth Weight: A Meta-Analysis Using Individual Participant Data**

**Review question(s)**

Low birthweight (LBW) may be the result of fetal growth restriction (FGR) and/or preterm birth (< 37 weeks gestation), and is associated with increased infant mortality rates and health problems in adulthood [[1](#_ENREF_1), [2](#_ENREF_2)]. In developing countries both malaria and macronutrient (protein-energy) malnutrition (underweight and overweight) are common in pregnancy and represent important risk factors for LBW [[3-5](#_ENREF_3)]. In the case of malaria, placental infection, in particular when associated with inflammation, is known to interfere with processes involved in securing optimal fetal growth such as transplacental nutrient transport [[6-8](#_ENREF_6)]. Although exact figures are unavailable and malaria prevalence has (temporarily) fallen in some countries, it is likely that the number of pregnancies simultaneously at risk of malnutrition and malaria is significant [[9](#_ENREF_9)]. Nutrition and infection are closely interlinked and one can lead to another [[10](#_ENREF_10), [11](#_ENREF_11)]. Effects of malnutrition on infection may be synergistic or antagonistic [[12](#_ENREF_12)].

Findings of a small number of clinical pregnancy studies conducted in malaria-endemic countries suggest that the deleterious impact of malaria on fetal growth and birthweight depends on women’s macronutrient nutritional status. In a Congolese cohort peripheral parasitaemia (*Plasmodium falciparum*) was associated with measuring small-for-gestational age (SGA, < 10^th^ centile; used as a indicator of FGR) only amongst undernourished women [[13](#_ENREF_13)]. Here, undernutrition was classified according to mid-upper arm circumference (MUAC < 23 cm) or body mass index (< 19.8 kg/m^2^) at first antenatal visit, both of which were independently associated with SGA in the cohort. In a sub-analysis of the same cohort early pregnancy malaria was also associated with abnormal utero-placental flow, but only amongst women with macronutrient undernutrition [[14](#_ENREF_14)]. Peripheral malaria was associated with reduced birth weight in undernourished women in Kenya (defined here as BMI for gestational age at first antenatal visit < 10^th^ centile) [[15](#_ENREF_15)]. However, both cohorts were comparatively small: this demands further evaluation of potential effect measure modification of the malaria-fetal size relationship by macronutrient nutritional status in larger studies [[16](#_ENREF_16)]. In cohort of pregnant women in Papua New Guinea (~2,000 women), macronutrient undernutrition exerted effect measure modification of the placental malaria-LBW relationship (Rogerson/Unger, manuscript in preparation): here, the risk of LBW associated with PM was highest in women with a normal MUAC, whereas PM did not further increase the risk of LBW in women who were undernourished. When using BMI as an indicator of undernutrition instead of MUAC effect measure modification was also observed, but in the opposite direction: the risk of LBW due to placental malaria was increased amongst women who were underweight. Taken together, these results suggest that maternal macronutrient status modifies the risk of FGR and LBW due to placental malaria, but the directionality and size of effect remains unclear, and may depend on the measures used to assess women’s nutritional status and to define malaria.

MUAC increases with gestational age, but only slightly and mostly in third trimester [[17](#_ENREF_17)]. This makes it a useful tool to identify pregnant women with macronutrient deficiency when a reliable gestational age is unknown [[18](#_ENREF_18), [19](#_ENREF_19)]: late presentation to ANC, lack of pre-pregnancy weight information, gestational weight gain and fluid retention have the potential to confound the nutrition and PM relationship when BMI is used. MUAC correlates strongly with BMI in non-pregnant adults, in particular at lower BMI ranges, and an MUAC < 23 cm is used by intensive care units in industrialised countries to detect macronutrient undernutrition in critically-ill patients for whom weight measurements are impractical [[20](#_ENREF_20)]. A low MUAC in pregnancy is associated with important adverse pregnancy outcomes including LBW and was deemed the most useful measure of nutritional status in pregnant women in the context of humanitarian emergencies and routine antenatal care in LMICs [[18](#_ENREF_18), [19](#_ENREF_19), [21-23](#_ENREF_21)].

Antenatal care can integrate interventions to address maternal malnutrition with malaria prevention such as intermittent preventive treatment in pregnancy (IPTp). Confirming effect measure modification of the malaria-LBW relationship by nutrition, assessed using MUAC or BMI, and understanding its directionality (synergism/antagonism) and size by evaluating a number of similar data sets together (weighted pooled analysis – meta analysis) may provide incentives to develop interventions to adequately address the likely twin burden of malaria and malnutrition as well as provide data that supports further research into the interplay between nutrition, infection and its impact on fetal growth.

**Types of study to be included**

We propose to pool data sets from pregnancy studies (cohort studies and randomized control trials) conducted in malaria-endemic areas (Africa, Asia and Oceania, South and Central America) to evaluate the interaction between maternal nutritional status and placental malaria in causing adverse pregnancy outcomes (LBW, reduced mean birthweight, and SGA, PTD in data sets with reliable estimates of gestation).

Potentially eligible studies/date sets will be identified through a comprehensive literature search, and key authors will be contacted regarding data sharing.

**Condition or domain being studied**

Malaria infection during pregnancy, malnutrition, adverse pregnancy outcomes

**Participants/ population**

*Studies will be eligible to contribute primary data to this meta-analysis if they fulfill the following criteria:*

- Ethical approval allows for secondary analyses and data sharing
- Able to access data set through original study team or publically available data
- Nutritional status assessed (pre-pregnancy or first antenatal MUAC and/or weight and height [BMI])
- Malaria measures at enrolment (first antenatal visit) available (light microscopy or qPCR)
- Infant weighed within 24 hours postpartum, or within 7 days of birth if timing of weight measurement data available.
- Age available
- Gravidity status available
- Malaria prevention clinical trial or observational study

*Participant exclusion criteria*

- Multiple pregnancy
- Miscarriage or stillbirth
- Birthweight not measured
- Nutritional status not evaluated at enrolment (or pre-pregnancy)
- Malaria status not evaluated at delivery
- Unknown gravidity
- Unknown infant sex
- Unknown IPTp/ISTp status

**Intervention(s), exposure(s)**

Malaria status first antenatal visit, by LM (positivity and species) and mid-upper arm circumference and/or BMI (pre-conception or at first antenatal visit). Different measures of nutrition (MUAC, BMI) and cut-offs to define undernutrition will be evaluated.

**Comparator(s)/ control**

Lack of a positive malaria diagnostics and no anthropometric indication of malnutrition.

**Context**

Studies conducted in malaria-endemic settings with medium to high transmission.

**Outcome(s)**

## Primary outcomes

Delivery weight (used to define LBW=weight <2,500 grams)

## Secondary outcomes

Preterm birth

Small-for-gestational age

**Data extraction, (selection and coding)**

The following variables will be selected for data abstraction by data managers using a standardized data transfer file:

Essential

- MUAC and/or BMI (pre-conception or at first antenatal visit)
- Gravidity (continuous variable)
- Age (continuous variable)
- Study type (malaria prevention or treatment) and details of regimens used.
- Birthweight, if measured > 24 hours, how long since birth?
- Infant sex
- Malaria status first antenatal visit, by LM (positivity and species)
- Rural/urban residence

Optional

- Haemoglobin at first antenatal visit
- Gestational age at enrolment (specify method that was used to establish GA)
- Fundal height
- Smoking
- Date of enrolment
- LLIN ownership
- GA at delivery (specify method used to establish GA) [Preterm birth]
- Neonatal anthropometric measures
  - Head circumference
  - Chest circumference
  - Abdominal circumference
  - Crown-heel length
- HIV status
- Malaria status first antenatal visit, by qPCR (positivity and species)
- Malaria status at delivery: One or more of the following
  - Peripheral parasitaemia, by LM
  - Placental infection, by LM
  - Placental histology

**Strategy for data synthesis**

Data will be pooled using random effect meta-analysis (weighed for study site and sample size). Risk ratios for the respective relationships of malnutrition and placental malaria will be calculated.

Risk ratios will be adjusted for a minimum set of potential confounders, namely includes ethnicity, malaria prevention (IPTp [type and number of treatment]/IST), haemoglobin at enrolment, gravidity, HIV infection and fetal sex.

Interaction terms will be fitted in inverse-probability of treatment weighted models. The effect of PM on LBW/SGA/PTD will be further evaluated through stratification according to nutritional status (using different criteria, e.g. MUAC, BMI). Forest plots will be used to visualise heterogeneity (quantified as $\tau^{2}$) between studies in terms of effects of malnutrition or PM on outcomes.

**Analysis of subgroups or subsets**

Subanalyses will be conducted for women with pre-pregnancy measures of nutrition. Sensitivity analyses will be performed e.g. by excluding studies that show strong high heterogeneity (quantified as $\tau^{2}$) in terms of effect of malnutrition or PM on birth outcomes as well as effect measurement modification. We will assess the influence of study characteristics on the heterogeneity of effect estimates using meta-regression. Specifically, we will evaluate the following study-level characteristics: time period of study (≤2007/2008-), study type (RCT/observational), and location (sub-Saharan Africa/ Oceania).

References

1. Kramer MS. Determinants of low birth weight: methodological assessment and meta-analysis. Bull World Health Organ **1987**;65(5):663-737.

2. Barker DJ, Gluckman PD, Godfrey KM, Harding JE, Owens JA, Robinson JS. Fetal nutrition and cardiovascular disease in adult life. Lancet **1993** Apr 10;341(8850):938-41.

3. Black RE, Victora CG, Walker SP, et al. Maternal and child undernutrition and overweight in low-income and middle-income countries. Lancet **2013** Aug 3;382(9890):427-51.

4. Grijalva-Eternod CS, Wells JC, Cortina-Borja M, et al. The double burden of obesity and malnutrition in a protracted emergency setting: a cross-sectional study of Western Sahara refugees. PLoS Med **2012**;9(10):e1001320.

5. Desai M, ter Kuile FO, Nosten F, et al. Epidemiology and burden of malaria in pregnancy. Lancet Infect Dis **2007** Feb;7(2):93-104.

6. Chandrasiri UP, Chua CL, Umbers AJ, et al. Insight into the pathogenesis of fetal growth restriction in placental malaria: decreased placental glucose transporter isoform 1 expression. J Infect Dis **2014** May 15;209(10):1663-7.

7. Umbers AJ, Boeuf P, Clapham C, et al. Placental malaria-associated inflammation disturbs the insulin-like growth factor axis of fetal growth regulation. J Infect Dis **2011** Feb 15;203(4):561-9.

8. Boeuf P, Aitken EH, Chandrasiri U, et al. Plasmodium falciparum malaria elicits inflammatory responses that dysregulate placental amino acid transport. PLoS Pathog **2013** Feb;9(2):e1003153.

9. Burki TK. Malaria and malnutrition: Niger's twin crises. Lancet **2013** Aug 17;382(9892):587-8.

10. Katona P, Katona-Apte J. The interaction between nutrition and infection. Clin Infect Dis **2008** May 15;46(10):1582-8.

11. Schaible UE, Kaufmann SH. Malnutrition and infection: complex mechanisms and global impacts. PLoS Med **2007** May;4(5):e115.

12. Clark MA, Goheen MM, Fulford A, et al. Host iron status and iron supplementation mediate susceptibility to erythrocytic stage Plasmodium falciparum. Nature communications **2014**;5:4446.

13. Landis SH, Lokomba V, Ananth CV, et al. Impact of maternal malaria and under-nutrition on intrauterine growth restriction: a prospective ultrasound study in Democratic Republic of Congo. Epidemiol Infect **2009** Feb;137(2):294-304.

14. Griffin JB, Lokomba V, Landis SH, et al. Plasmodium falciparum parasitaemia in the first half of pregnancy, uterine and umbilical artery blood flow, and foetal growth: a longitudinal Doppler ultrasound study. Malar J **2012** Sep 10;11(1):319.

15. McClure EM, Meshnick SR, Lazebnik N, et al. A cohort study of Plasmodium falciparum malaria in pregnancy and associations with uteroplacental blood flow and fetal anthropometrics in Kenya. Int J Gynaecol Obstet **2014** Jul;126(1):78-82.

16. Ioannidis JP. Why most published research findings are false. PLoS Med **2005** Aug;2(8):e124.

17. Lopez LB, Calvo EB, Poy MS, del Valle Balmaceda Y, Camera K. Changes in skinfolds and mid-upper arm circumference during pregnancy in Argentine women. Matern Child Nutr **2011** Jul;7(3):253-62.

18. Liljestrand J, Bergstrom S. Antenatal nutritional assessment: the value of upper arm circumference. Gynecologic and obstetric investigation **1991**;32(2):81-3.

19. Ververs MT, Antierens A, Sackl A, Staderini N, Captier V. Which anthropometric indicators identify a pregnant woman as acutely malnourished and predict adverse birth outcomes in the humanitarian context? PLoS Curr **2013**;5.

20. Ravasco P, Camilo ME, Gouveia-Oliveira A, Adam S, Brum G. A critical approach to nutritional assessment in critically ill patients. Clin Nutr **2002** Feb;21(1):73-7.

21. Collins S. Using middle upper arm circumference to assess severe adult malnutrition during famine. JAMA **1996** Aug 7;276(5):391-5.

22. Collins S, Duffield A, Myatt M. Assessment of nutritional status in emergency-affected populations - adults. Geneva: United Nations, **2000**.

23. Collins S, Myatt M. Short-term prognosis in severe adult and adolescent malnutrition during famine: use of a simple prognostic model based on counting clinical signs. JAMA **2000** Aug 2;284(5):621-6.

24. WHO. BMI Classification. Available at: http://apps.who.int/bmi/index.jsp?introPage=intro_3.html. Accessed 21 Sep 12.

25. Brabin L, Brabin BJ, Gies S. Influence of iron status on risk of maternal or neonatal infection and on neonatal mortality with an emphasis on developing countries. Nutr Rev **2013** Aug;71(8):528-40.

26. Senga EL, Harper G, Koshy G, Kazembe PN, Brabin BJ. Reduced risk for placental malaria in iron deficient women. Malar J **2011**;10:47.

27. Jonker FA, Boele van Hensbroek M, Leenstra T, et al. Conventional and novel peripheral blood iron markers compared against bone marrow in Malawian children. J Clin Pathol **2014** Aug;67(8):717-23.

28. van den Broek NR, Letsky EA. Etiology of anemia in pregnancy in south Malawi. Am J Clin Nutr **2000** Jul;72(1 Suppl):247S-56S.
