# Supplementary figures and images for: Malaria, malnutrition, and birthweight: A meta-analysis using individual participant data
Source: PLoS Med. 2017 Aug 8;14(8):e1002373. doi: 10.1371/journal.pmed.1002373 (PMC5549702; doi:10.1371/journal.pmed.1002373)

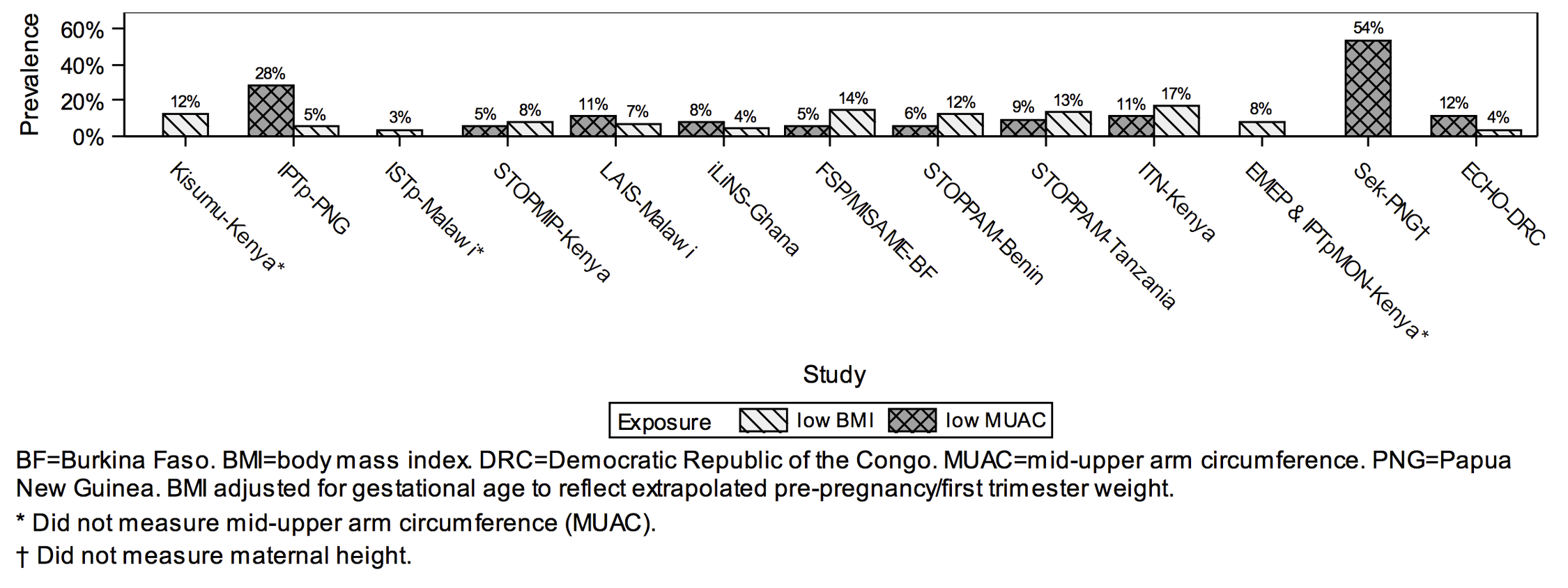

Supplement: S1 Fig — (DOCX) [file pmed.1002373.s011.docx]

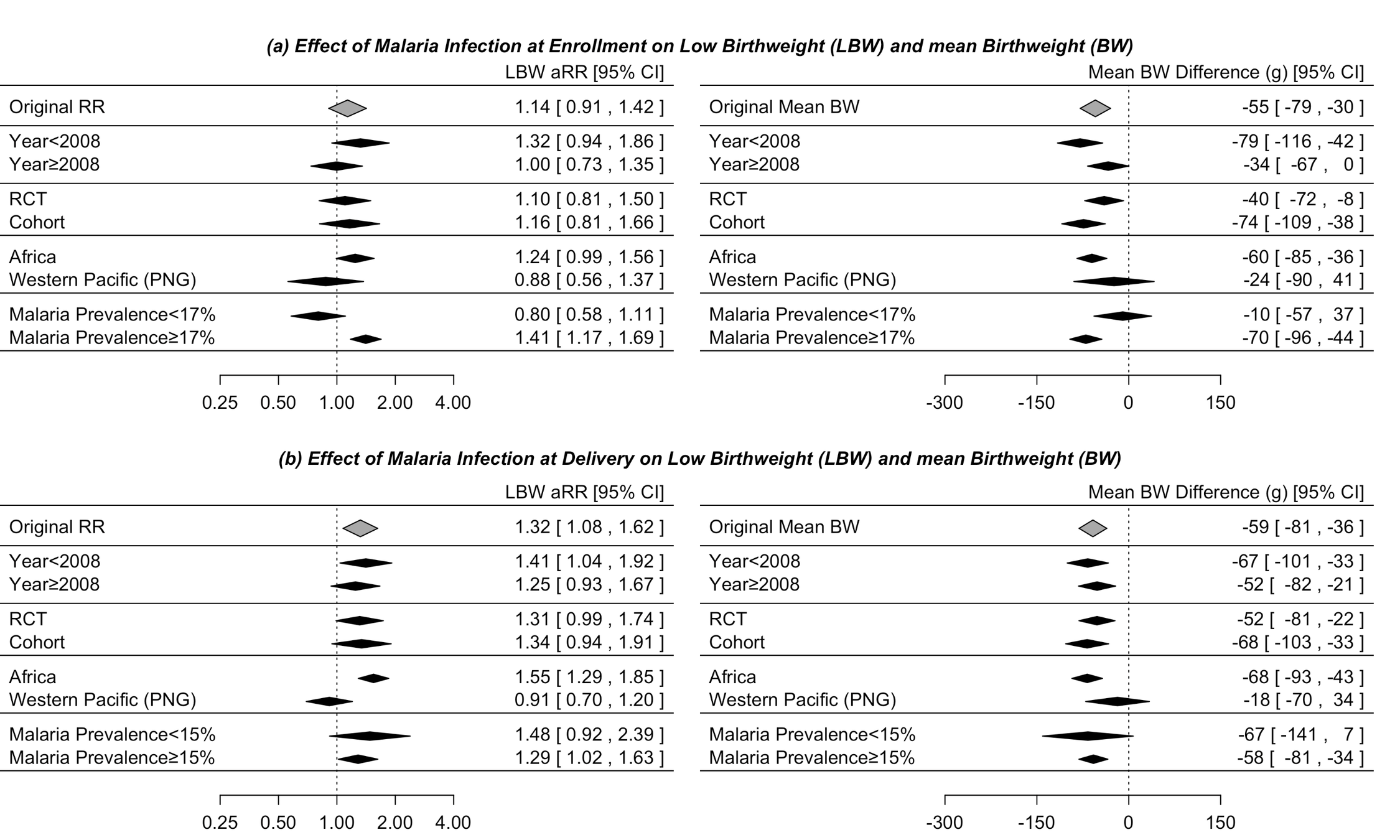

Supplement: S2 Fig — Median malaria prevalence across studies was 17% at enrollment and 15% at delivery. RCT = randomized control trial. (DOCX) [file pmed.1002373.s012.docx]

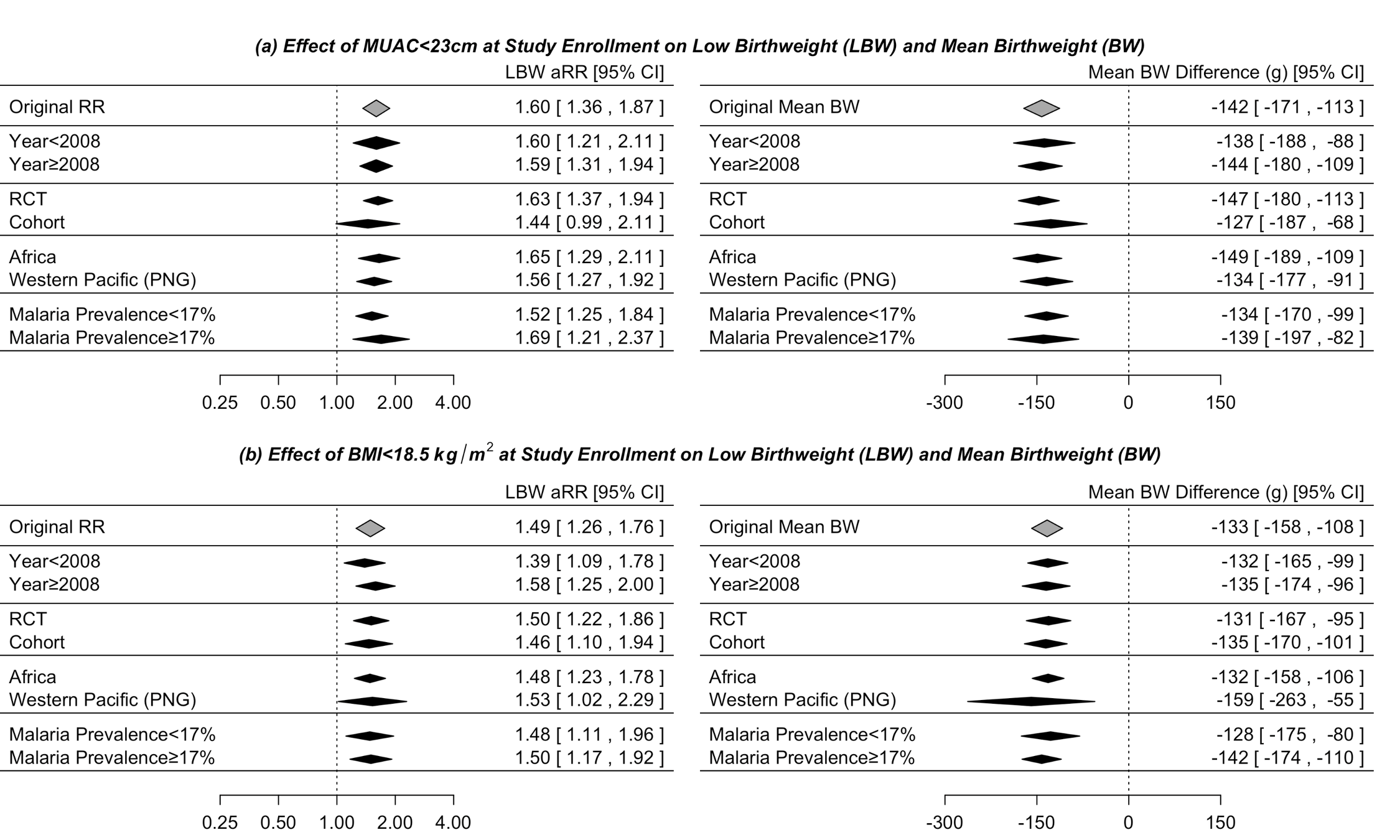

Supplement: S3 Fig — Median malaria prevalence across studies was 17% at enrollment and 15% at delivery. RCT = randomized control trial. (DOCX) [file pmed.1002373.s013.docx]

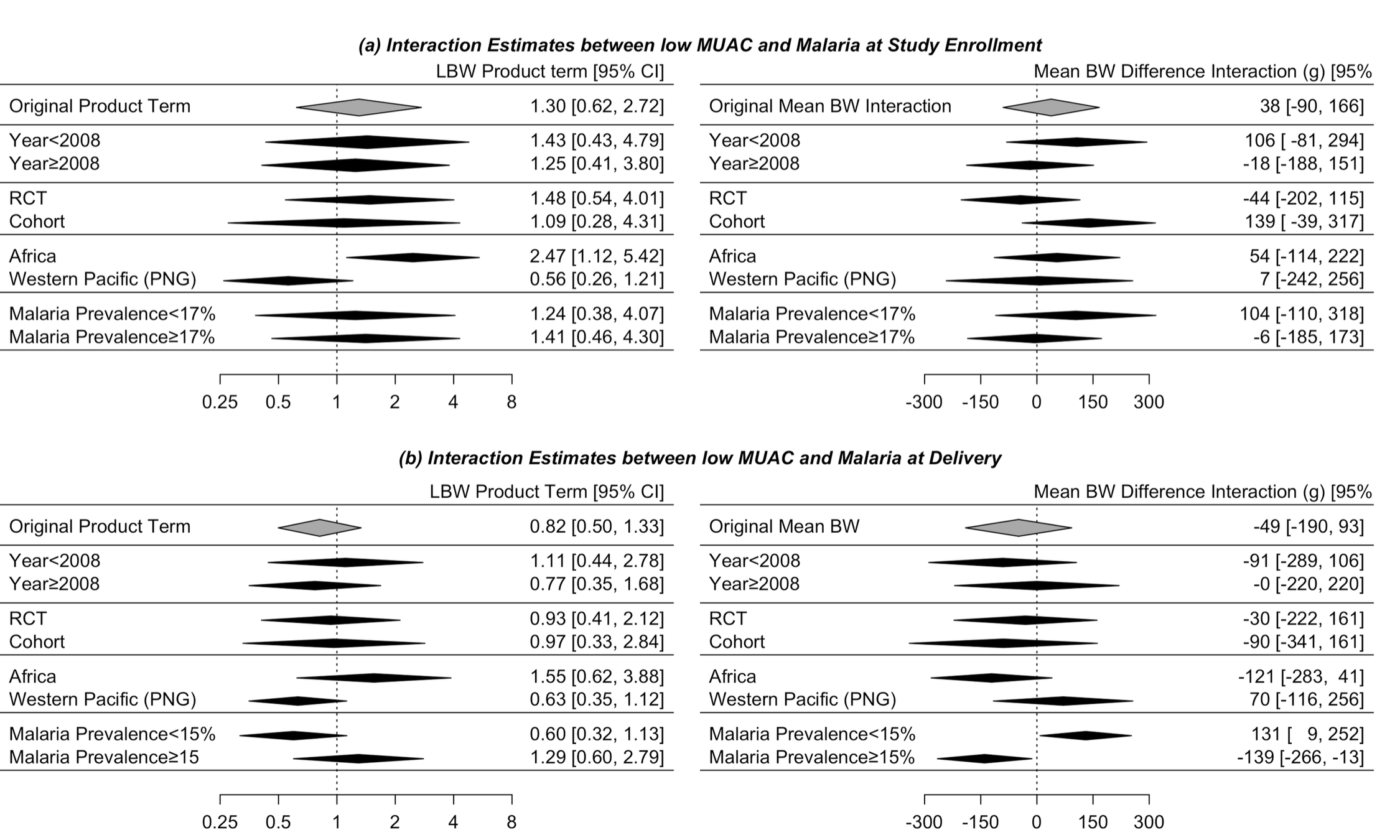

Supplement: S4 Fig — Median malaria prevalence across studies was 17% at enrollment and 15% at delivery. (DOCX) [file pmed.1002373.s014.docx]

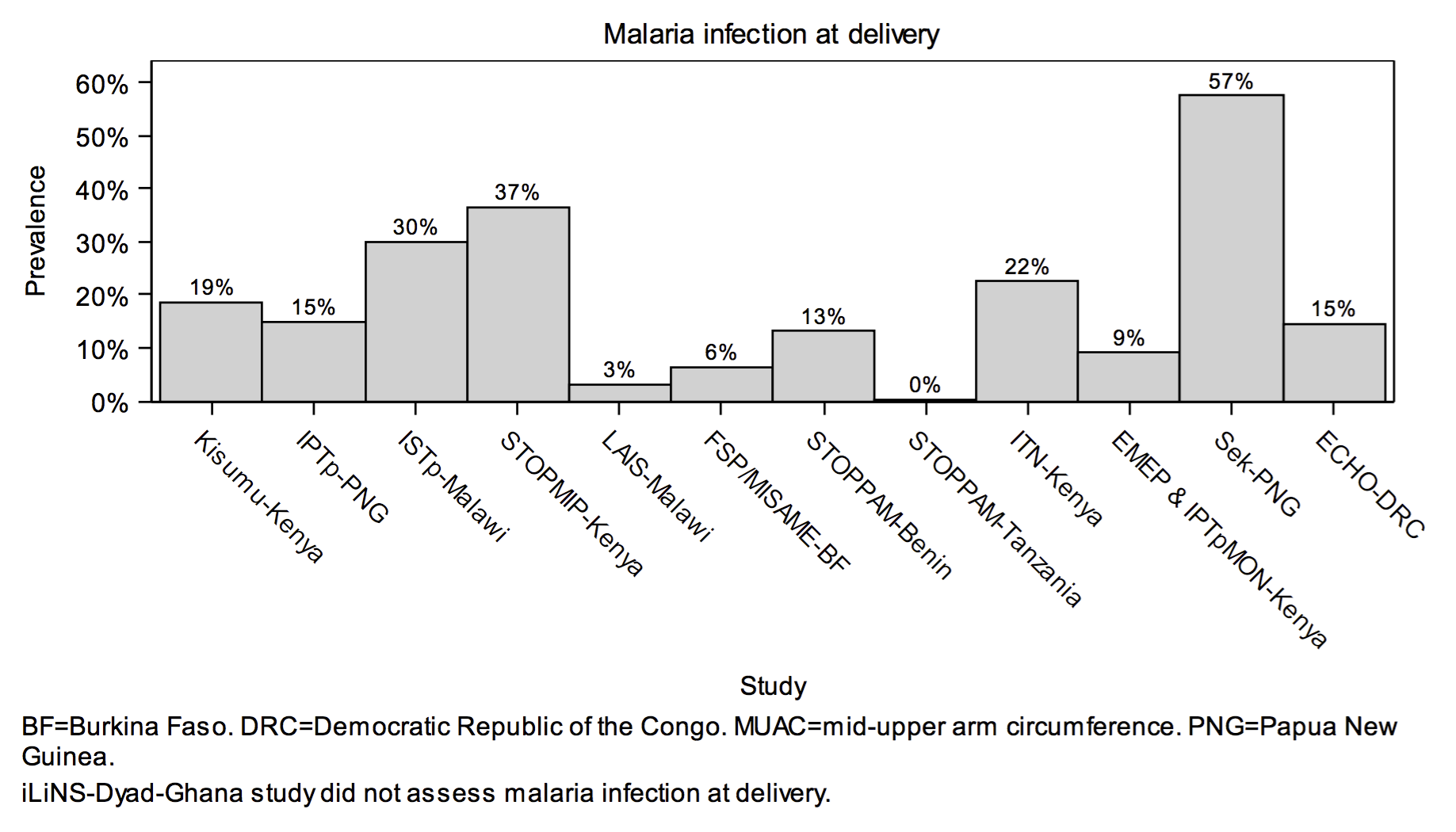

Supplement: S5 Fig — (DOCX) [file pmed.1002373.s015.docx]
